# Postsynaptic density radiation signature following space irradiation

**DOI:** 10.3389/fphys.2023.1215535

**Published:** 2023-06-27

**Authors:** Soren Impey, Carl Pelz, Lara-Kirstie Riparip, Amanuel Tafessu, Fatema Fareh, Damian G. Zuloaga, Tessa Marzulla, Blair Stewart, Susanna Rosi, Mitchell S. Turker, Jacob Raber

**Affiliations:** ^1^ Department of Pediatrics, Oregon Stem Cell Center, Oregon Health and Science University, Portland, OR, United States; ^2^ Dow Neuroscience Laboratories, Department of Cell and Developmental Biology, Legacy Research Institute, Legacy Health Systems, Oregon Health and Science University, Portland, OR, United States; ^3^ Departments of Neurological Surgery and Physical Therapy and Rehabilitation Science, Brain and Spinal Injury Center, University of California, San Francisco, San Francisco, CA, United States; ^4^ Department of Behavioral Neuroscience, Oregon Health and Science University, Portland, OR, United States; ^5^ Department of Molecular and Medical Genetics, Oregon Institute of Occupational Health Sciences, Oregon Health and Science University, Portland, OR, United States; ^6^ Departments of Neurology and Radiation Medicine, Division of Neuroscience ONPRC, Oregon Health and Science University, Portland, OR, United States

**Keywords:** postsynaptic, DNA methylation, hippocampus, object recognition, space radiation

## Abstract

**Introduction:** The response of the brain to space radiation is an important concern for astronauts during space missions. Therefore, we assessed the response of the brain to ^28^Si ion irradiation (600 MeV/n), a heavy ion present in the space environment, on cognitive performance and whether the response is associated with altered DNA methylation in the hippocampus, a brain area important for cognitive performance.

**Methods:** We determined the effects of ^28^Si ion irradiation on object recognition, 6-month-old mice irradiated with ^28^Si ions (600 MeV/n, 0.3, 0.6, and 0.9 Gy) and cognitively tested two weeks later. In addition, we determined if those effects were associated with alterations in hippocampal networks and/or hippocampal DNA methylation.

**Results:** At 0.3 Gy, but not at 0.6 Gy or 0.9 Gy, ^28^Si ion irradiation impaired cognition that correlated with altered gene expression and 5 hmC profiles that mapped to specific gene ontology pathways. Comparing hippocampal DNA hydroxymethylation following proton, ^56^Fe ion, and ^28^Si ion irradiation revealed a general space radiation synaptic signature with 45 genes that are associated with profound phenotypes. The most significant categories were glutamatergic synapse and postsynaptic density.

**Discussion:** The brain’s response to space irradiation involves novel excitatory synapse and postsynaptic remodeling.

## 1 Introduction

A unique feature of the space radiation environment is the presence of galactic cosmic rays (GCR) and solar particle events (SPE) (Kronenberg and Cucinotta, 2012). The former involves protons and fully ionized atomic nuclei such as ^28^Si, while the latter includes predominantly low to medium energy protons with a small heavy ion component. Space irradiation exposures pose a significant health hazard to space flight crews during missions (Ferlazzo and Foray, 2017) but uncertainty exists with respect to the level of risk to develop cognitive changes following irradiation doses relevant to those encountered during space missions and there are individual difference of risk as well to consider (Foray et al., 2016; Restier-Verlet et al., 2021). In the central nervous system (CNS), radiation exposure affects the hippocampus (for a review, see Kiffer et al., 2019), a structure critical for memory function. We reported that object recognition memory (Ennaceur, 2010), which uses a 24-h interval between learning and memory assessment to test hippocampal function (Raber, 2015), is impaired 2 weeks following irradiation of 6-month-old mice with ^56^Fe ions (600 MeV/n, 0.1 Gy) (Impey et al., 2016a) and protons (150 MeV, 1 Gy) and associated with alterations in hippocampal DNA methylation (Impey et al., 2016b).

The mechanisms mediating the effects of simulated space irradiation on hippocampus-dependent cognitive function might be associated with changes in hippocampal networks. We earlier reported ^56^Fe ion- and proton-irradiation related changes in immediate early gene Activity-Regulated Cytoskeleton-Associated Protein (*Arc*) in the hippocampus (Penner et al., 2010; Impey et al., 2016a; Impey et al., 2016b).

The mechanisms mediating the effects of simulated space irradiation on hippocampus-dependent cognitive function might be associated with alteration in hippocampal DNA methylation as well. Changes in cytosine methylation involving the addition of a methyl group to cytosine (5 mC and especially those involving addition of a hydroxy group to 5 mC (hydroxymethylcytosine or 5 hmC) (Veron and Peters, 2011) play a key role in regulating expression of genes required for learning and memory (Miller and Sweatt, 2007; Lubin et al., 2008). Hippocampal DNA methylation is affected following proton (Impey et al., 2016b) and ^56^Fe irradiation (Impey et al., 2016a).

In the current study, to determine the effects of ^28^Si ion irradiation on object recognition, 6-month-old mice were irradiated with ^28^Si ions (600 MeV/n, 0.3, 0.6, an 0.9 Gy) and cognitively tested 2 weeks later. In addition, we determined if those effects were associated with alterations in hippocampal networks and/or hippocampal DNA methylation.

## 2 Materials and methods

### 2.1 Animals and study design

Six-month-old C57BL/6J male mice (*n* = 84 mice in total) were obtained from Jackson Laboratories, Bar Harbor Maine. The biological age of the mice was selected to be relevant to the biological age of astronauts during space missions. The mice were shipped from Jackson Laboratories to Brookhaven National Laboratory (BNL), Upton, New York, and allowed to accommodate to the housing facility there for 1 week. Subsequently, the mice were irradiated with 0.3, 0.6, or 0.9 Gy of 600 MeV ^28^Si ion or sham-irradiated (*n* = 42 mice/radiation dose). The duration of the radiation exposures was 0.99 ± 0.08 min, 1.53 ± 0.08 min, and 2.10 ± 0.07 min, for the 0.3, 0.6, and 0.9 Gy dose respectively. For irradiation, mice were individually loaded into 8 × 3 × 3 cm plastic square enclosures with air holes and placed in a foam fixture in the beam line of the NASA Space Radiation Laboratory (NSRL). They were exposed to a rectangular beam of approximately 20 × 20 cm. The focused beam of high-energy was generated by the Booster accelerator at BNL and transferred to the experimental beam line at the NSRL facility. Dose calibration was performed so that the desired dose could be delivered. Sham-irradiated mice were placed into the plastic enclosures for the same time as the irradiated mice. Mice were randomly assigned to the experimental groups. The week after the irradiation or sham-irradiation, the mice were shipped to Oregon Health & Science University (OHSU) and cognitive testing started 2 weeks following irradiation. Mice were tested in the open field and for novel object recognition in week 1. In week 3, mice were tested for hippocampal network stability and euthanized immediately after by cervical dislocation. The hippocampus of one hemibrain was dissected for DNA methylation and RNAseq analyses. The other hemibrain was frozen in −70°C isopentane (2-methyl butane, Sigma) and processed for *Arc* mRNA and TET2 immunohistochemical analyses. The hemibrains were blocked together such that each slide contained sections from different experimental groups. All slides were cryosectioned and stored at −70°C until processed for immunocytochemistry or fluorescence *in situ* hybridization (FISH). All protocols were reviewed and approved by the Institutional Animal Care and Use Committees (IACUC) of OHSU and BNL and were in compliance with all Federal regulations.

### 2.2 Novel object recognition

The novel object recognition test was performed as described (Raber et al., 2002). The mice were habituated to an open field (16 × 16 inches, Kinder Scientific, Poway, CA) for 3 times for 10 min each over three subsequent days. On day 4, the mice were placed in the open field containing two identical objects and they were allowed to freely explore for 15 min. On day 5, the mice were placed again in the open field, but one familiar object was replaced with a novel object. The mice were allowed to explore for 15 min. Movement and time spent exploring each object was recorded using Ethovision XT video tracking system (Noldus Information Technology, Sterling, VA) and hand scored by a researcher blinded to the treatment of the mice. The percent time exploring the novel object, out of the total time exploring the novel and familiar objects on day 5, was used to assess novel object recognition. For each group, the preference for the novel versus the familiar object was assessed. The open field arena and objects were cleaned with 5% acetic acid between mice and trials.

### 2.3 Hippocampal network stability

Exploration of identical or different environments was used to study the stability of hippocampal networks (Guzowski et al., 1999), using a method called catFISH (cellular compartment analysis of temporal activity using fluorescence *in situ* hybridization) that relies on the precise temporal kinetics of the IEG and neuronal gene *Arc*, which is involved in synaptic plasticity and memory, as described previously (Impey et al., 2017). When neurons are engaged in information processing, *Arc* is rapidly transcribed and can be visualized and quantified after ∼5 min. Subsequently, the mRNA is translocated to the cytoplasm where it remains detectable for ∼20–30 min after the initial transcription. The mRNA is translocated to tagged synapses for protein synthesis. Two different cellular compartments (nuclear and cytoplasmic) can be clearly distinguished, allowing identification of neurons active during distinct behavioral experiences (Guzowski et al., 1999).

Eight mice from each experimental radiation condition were placed individually into a novel environment (A; a square open field; 61 × 61 cm box with 20-cm high walls) and allowed to explore for 5 minutes. Mice were returned to their cage for 25 min, returned to the same environment for an additional 5 min (AA Paradigm). Another 8 mice per radiation dose were allowed to explore environment A for 5 min, and 25 min later they were placed in a different environment (B; a circular arena 45 cm in diameter, and allowed to explore for 5 min; AB Paradigm). Following the last environmental exposure, the mice were killed by cervical dislocation and the brains quickly removed, as described above.

### 2.4 Fluorescence *in situ* hybridization

Four to six slides from each treatment group, each containing sections from all the mice in that group, were prepared for fluorescence *in situ* hybridization. Arc mRNA were detected as previously reported in detail (Rosi et al., 2009; Ramirez-Amaya et al., 2013). Briefly, hapten-labeled antisense riboprobes were hybridized together with the tissues overnight. The digoxigenin-labeled Arc full-probe riboprobe was detected with anti–digoxigenin-HRP conjugate (Roche) and revealed with a cyanine-3 (CY3) substrate kit. Nuclei were counterstained with sytox-green (Molecular Probes).

### 2.5 Microscopy, image acquisition, and analysis

Microscopic imaging for Arc mRNA, was performed using a Zeiss AXIO IMAGER Z1 microscope with motorized Z-drive for transmitted light and epifluorescence (Rosi et al., 2009). For each end point, the four to six coronal sections per mouse were used to image CA1 and CA3.

### 2.6 Image analysis

Manual cell counts of cells expressing Arc mRNA were performed by an experimenter blind to the relationship between the experimental conditions they represented. Cytoplasmic Arc mRNA–positive neurons were identified when the staining constituted at least 60% of the cell body (Rosi et al., 2009) and was detectable throughout three planes across the Z-stack. To avoid classification errors, we carefully verified that the staining belonged to the cell of interest by checking the nuclear counterstaining (Rosi et al., 2009). The neuronal nuclei were classified as follows: negative (no staining), Arc mRNA foci positive (containing only Arc intranuclear foci staining detected with the Arc intron probe), Arc mRNA cytoplasmic positive (containing only cytoplasmic staining detected with the full-length probe) (Arc-cyto), or as double-labeled for Arc pre-mRNA foci and Arc mRNA cytoplasmic (containing both intranuclear Arc pre-mRNA foci and cytoplasmic Arc mRNA transcripts in two different colors). When a count included all classifications we refer to it as an Arc-positive neuron (this includes the total number of the three above mentioned classifications). The percentages of Arc-foci, Arc-cyto, or Arc-double were calculated relative to the total number of pyramidal neurons included in the analysis as previously reported (Rosi et al., 2009).

### 2.7 Tet2 immunohistochemistry

Hemibrains were processed for Tet2 immunoreactivity using a specific primary antibody from Santa Cruz Biotechnology (Tet2 S-13, 1:250, catalog number sc-136926) and donkey anti-rabbit Alexa 488 (1:200) as secondary antibody and *n* = 3 sections per hemibrain, approximately 200 µm apart, per mouse, as described (Impey et al., 2017). Background threshold levels were set and applied to all images. Pixel intensities above this threshold were used for quantification measures (area occupied by pixels and intensities of pixels). The total intensity was also quantified as a measure of overall pixel intensity within a specific brain region.

### 2.8 DNA methylation

DNA was isolated from the hippocampus. Antibodies against 5 mC and 5 hmC were used to immunoprecipitate sonicated DNA preparations for methyl-DNA immunoprecipitation (meDIP--anti-5mC mouse mAb, EMD-Millipore NA8; catalog number 162 33 D3) and hydroxymethyl-DNA immunoprecipitation (hmeDIP; anti-5hmC rabbit polyclonal; catalog number 39769; 2 ul, Active Motif)), respectively, and Dynal anti-mouse IgG beads from pools of tissues. Beads were rinsed 7 times with IP buffer, eluted with 1% SDS at room temperature and the eluted DNA purified and subjected to limited amplification (∼18 cycles). Libraries were sequenced on the HiSeq2000 platform at the OHSU Massively Parallel Sequencing Shared Resource or the Oregon State University Center for Genome ReseArch. DIP-Seq regions methylated above “background” were identified using a sliding window method and enriched regions selected via a Monte Carlo-permutation test (Fejes et al., 2008).

### 2.9 RNAseq

To facilitate direct comparison of DIP-Seq data with gene expression data, RNA-Seq was used to profile transcription from the same animals used for the DIP-Seq experiments. RNA was isolated using the NEBnext poly A selection kit (New England Biolabs). Illumina high-throughput sequencing technology was used to profile RNA levels in an unbiased manner. For Illumina RNA-Seq library preparation, NEBnext Ultra kit was used according to the manufacturers specifications (New England Biolabs). Libraries were sequenced on the HiSeq2000 platform at the OHSU Massively Parallel Sequencing Shared Resource. Illumina data were mapped to the UC Santa Cruz assembly using Bowtie (Langmead et al., 2009). For RNA-Seq analyses, tags that overlap with known RefSeq gene models (UCSC RefSeq annotation) were counted using R scripts (Gentleman et al., 2004). Significance was assessed using the DESeq2 package (Love et al., 2014). The Storey Q-test was used to adjust for multiple comparisons (Storey and Tibshirani, 2003).

### 2.10 Bioinformatics and statistical analyses

The cognitive and *Arc* data are shown as mean ± SEM. The statistical analyses of the data were performed using SPSS™ (Chicago, IL) and GraphPad Prism™ (San Diego, CA) software packages. To analyze locomotor activity over 3 days, ability to locate a visible platform over 2 days, and ability to locate a hidden platform over 3 days, repeated measures ANOVA was used. To compare exploration of the objects and the percentage of *Arc*-positive cells and total number of *Arc* cells following exposure to the two different environmental conditions, 2-tailed *t*-tests were used. The cognitive and *Arc* figures were generated using GraphPad Prism software. We considered *p* < 0.05 as statistically significant.

Single read sequence data was mapped to the mouse reference genome (UCSC mm9) using the Bowtie algorithm using standard flags and allowing 2 mismatches (Langmead et al., 2009). Sequences that map to a single location were selected and domains enriched for 5 mC or 5 hmC were selected using a parameter-optimized Monte-Carlo-based segmentation algorithm (Fejes et al., 2008). A 1000 bp sliding-window was used based on iterative analyses that maximized the number of enriched regions. A comparison of different high-throughput sequencing based methods to study DNA methylation concluded that MeDIP-Seq covers ∼67% of genomic CpGs (Harris et al., 2010).

For statistical comparisons of biological samples, regions of methylation enrichment were merged and differences in methylation interrogated with FDR-adjusted negative binomial statistics (Anders and Huber, 2010). Statistical and visualization studies involved the R programming language and Bioconductor packages (Gentleman et al., 2004). Gene ontology analyses utilized the Bioconductor Goseq package, which adjusts for sequence-length bias artifacts in genomic data (Young et al., 2010). KEGG analyses were conducted using the DAVID-EASE site (Dennis et al., 2003). For gene ontology analyses the top DMRs (differentially methylation regions) or DHRs (differentially hydroxymethylated regions) (*p* < 0.01) within a 50 kb window centered on the RefSeq transcriptional start site or within the RefSeq gene body were non-redundantly annotated. Unless otherwise stated, overlap between DMRs and RNA-Seq data was analyzed using a similar windowing approach. For statistical comparisons of gene-annotated DHRs with DHRs or RNA-Seq gene data we used the Fisher Exact test (“stats” R core library) or for multi-set comparisons (>2 sets) the SuperExactTest R package (Wang et al., 2015). For Fisher Exact tests FDR-adjusted *p* < 1 × 10^−3^ was considered significant.

DIP sequence-tag heatmaps were generated in R by plotting median-normalized DIP-Seq tag density in gene bodies and indicated flanking regions with color-maps scaled to the 80% quantile. Statistical analyses of pathway data were conducted using FDR-adjusted Fisher exact test. Statistical analyses of DHR density in genomic regions were conducted using a Monte-Carlo-based permutation statistic (“coin” R package). Unless otherwise stated, FDR-adjusted *p* < 0.01 was considered statistically significant.

## 3 Results

### 3.1 Open field and novel object recognition

**FIGURE 1:**
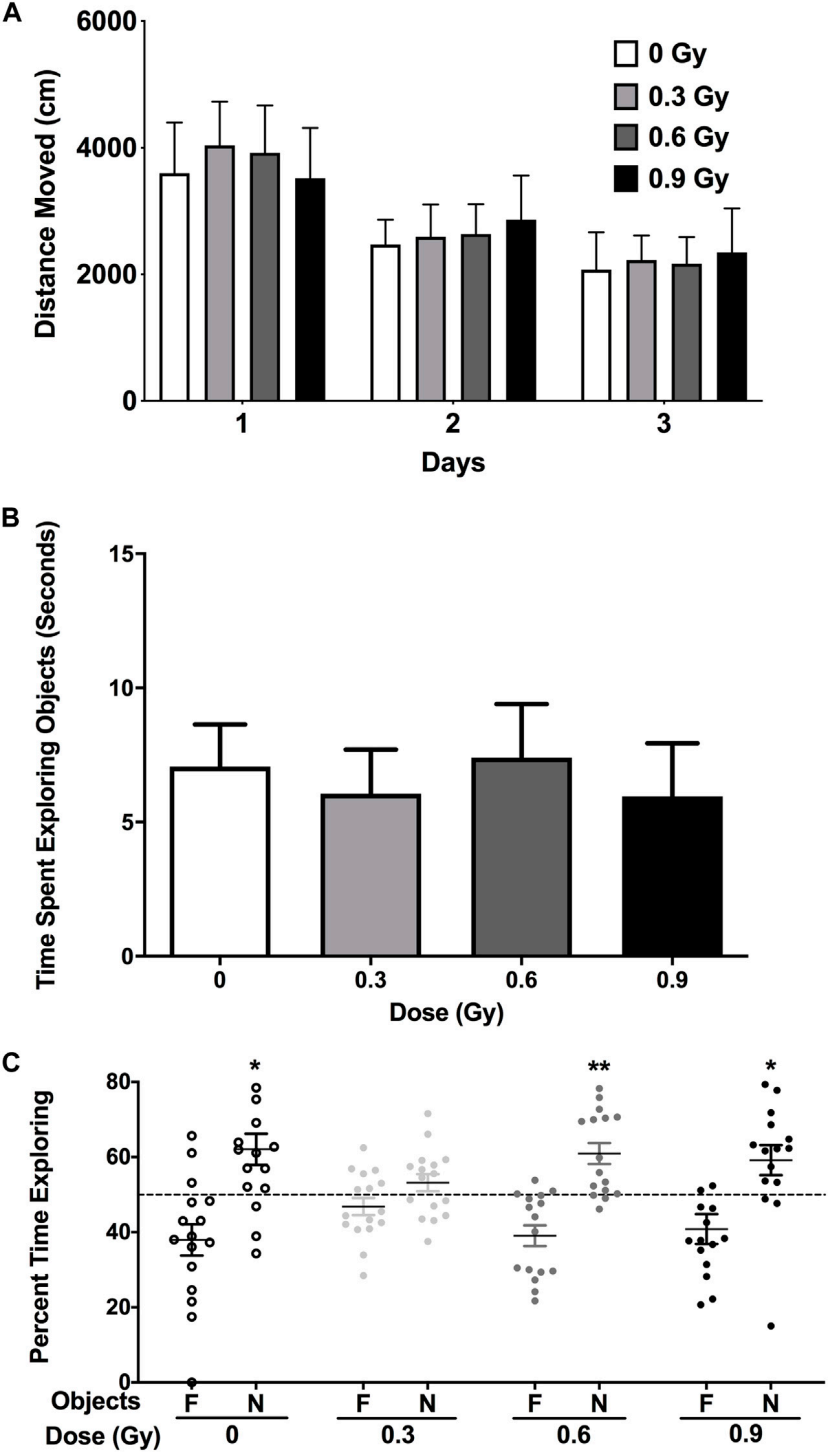
**(A)** ^28^Si ion irradiation did not affect activity levels in the open field on three subsequent day. **(B)** Object recognition. There was no effects of radiation on the time the mice spent exploring the objects. **(C)** Sham-irradiated mice and mice irradiated with a dose of 0.6 or 0.9 Gy showed object recognition and spent significantly more time exploring the novel than the familiar object. In contrast, mice irradiation with a dose of 0.3 Gy did not. *N* = 16 mice/dose. *p < 0.05, **p < 0.01 versus familiar object. N: novel object; F: familiar object.

The mice were first habituated to an open field for three subsequent days. ^28^Si ion irradiation did not affect activity levels in the open field over these 3 days (Figure 1A). On day 4, the mice were exposed to the open field containing two objects for 15 min. On day 5, the mice were exposed to the open field with two objects for 15 min, but one familiar object (present on day 4) was replaced with a novel one. There was no effects of radiation on the time the mice spent exploring the objects (Figure 1B). The percent time exploring the novel object was used to assess novel object recognition. Following ^28^Si (600 MeV) ion irradiation, mice irradiated with 0.3 Gy were impaired, as revealed by no discrimination between the familiar and novel object, while those irradiated with higher doses were not (Figure 1C).

### 3.2 Hippocampal network activity

**FIGURE 2:**
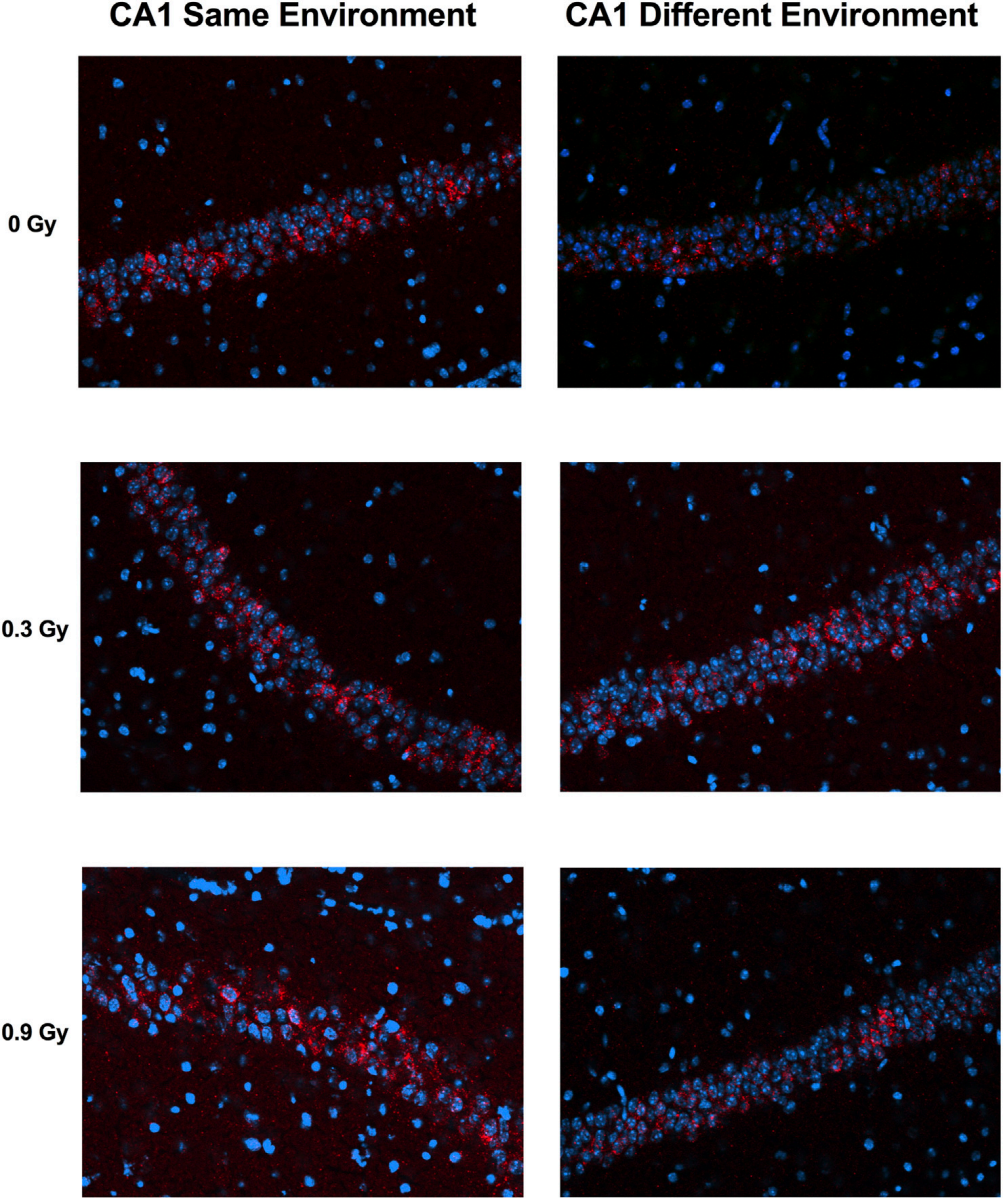
Representative images for Arc catFISH data in the CA1 region of the hippocampus. Representative fluorescence images showing Arc mRNA expression following exploration of the same or different environments (images taken at 20 Å∼ 1 z stack). Scale bar: 100 μm. The Arc mRNA is illustrated in red and cell nuclei are indicated in blue (DAPI).

**FIGURE 3:**
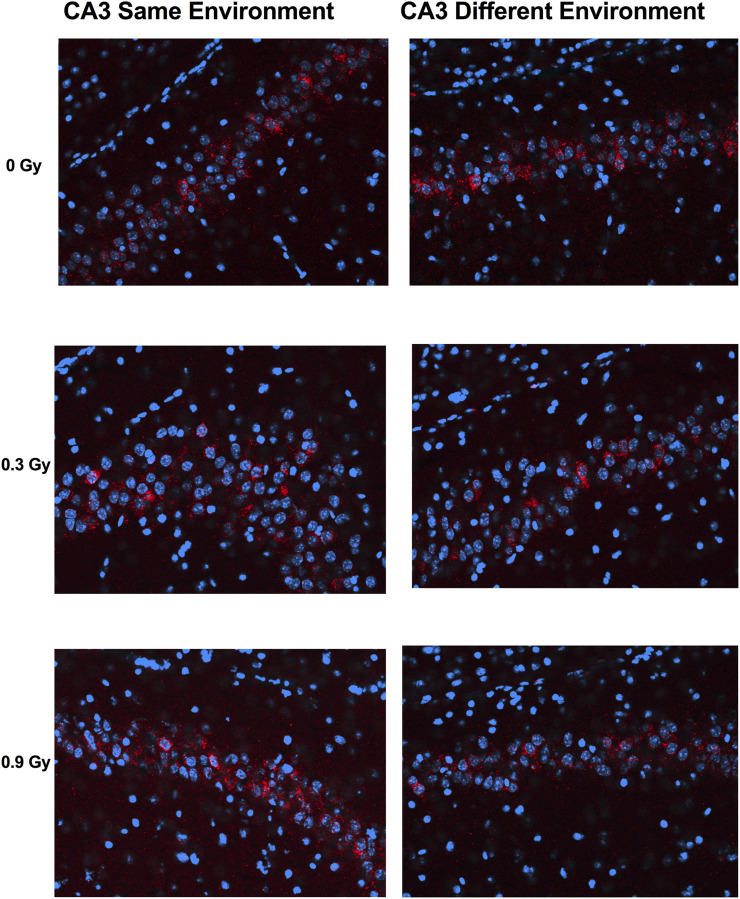
Representative images for Arc catFISH data in the CA3 region of the hippocampus. Representative fluorescence images showing Arc mRNA expression following exploration of the same or different environments (images taken at 20 Å∼ 1 z stack). Scale bar: 100 μm. The Arc mRNA is illustrated in red and cell nuclei are indicated in blue (DAPI).

**FIGURE 4:**
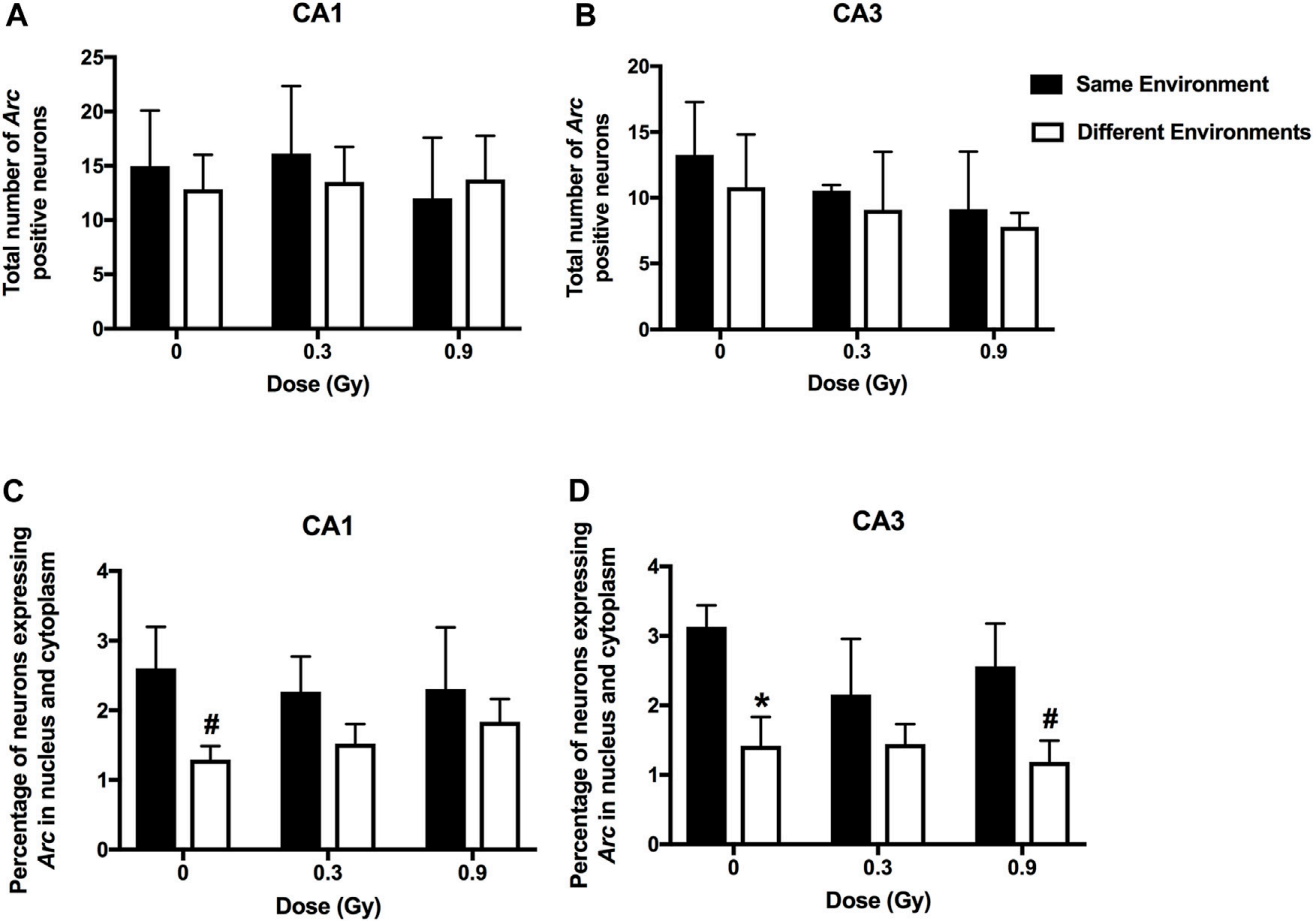
A comparable total number of *Arc*-positive neurons in the CA1 **(A)** and CA3 **(B)** regions of the hippocampus of sham-irradiated mice and mice irradiated with 0.3 or 0.9 Gy of ^28^Si ions. **(C)** There was a trend towards a higher percentage of *Arc*-positive neurons in the CA1 region of the hippocampus of sham-irradiated mice following exposure to twice the same environment than two different environments. This was not seen in mice irradiated with 0.3 or 0.9 Gy. **(D)** In sham-irradiated mice, the percentage of *Arc*-positive neurons expressing *Arc* mRNA in the nucleus and cytoplasm in the CA3 region of the hippocampus following exposure twice to the same environment was higher than following exposure to two different environments and there was a trend towards a higher percentage of *Arc*-positive neurons in the CA3 region of the hippocampus of mice irradiated with a dose of 0.9 Gy following exposure to twice the same environment than two different environments. *p = 0.011 versus same environment; ^#^ *p* = 0.05. CA1; *N* = 4–7 mice/dose/environment; CA3: *N* = 3–6 mice/dose/environment. Due to technical problems, tissues from mice irradiated with a dose of 0.6 Gy could not be analyzed.

We next assessed whether the mechanisms mediating the cognitive effects of ^28^Si ion irradiation might be associated with reduced ability of hippocampal neurons to recognize similar environments and to discriminate distinct environments using the temporal kinetics of mRNA encoding *Arc*. Exploratory behavior activated a comparable total number of neurons in sham-irradiated and irradiated mice in the CA1 (Figures 2, 4A) and CA3 (Figures 3, 4B) regions of the hippocampus. However, there was an effect of ^28^Si ion irradiation on the percent of neurons activated by both experiences. The percentage of *Arc*-positive neurons expressing *Arc* mRNA in the nucleus and cytoplasm in the CA3 region of the hippocampus of sham-irradiated mice was significantly higher following exposure twice to the same environment, as opposed to exposure to two different environments (*t* = 3.191, *p* = 0.0110, Figure 4D) and there was a trend towards a difference in the CA1 region of the hippocampus of sham-irradiated mice (*t* = 2.108, *p* = 0.0567, Figure 4C). This was not seen in mice irradiated with 0.3 Gy that showed impaired cognitive performance (CA1: *t* = 1.286, *p* = 0.2273, Figure 3C; CA3: *t* = 1.051, *p* = 0.3280; Figure 2D). In mice irradiated with 0.9 Gy, who showed no impaired cognitive performance (Figure 1B), there was a trend towards a difference in the CA3 region (*t* = 2.237, *p* = 0.00557, Figure 4D), but not the CA1 region (*t* = 0.5842, *p* = 0.5752, Figure 4C).

### 3.3 Hippocampal DNA methylation

We next determined whether ^28^Si ion irradiation-induced cognitive injury is associated with pathway changes in the hippocampus by comparing hippocampal pathway changes in mice irradiated at a lower dose that caused cognitive injury with hippocampal pathway changes in mice irradiated at a higher dose that did not cause cognitive injury. We did not see effects of ^28^Si ion irradiation on TET2 immunoreactivity in the dentate gyrus, CA1 or CA3 region of the hippocampus, or cortex. The levels of 5 mC and 5 hmC levels are high and exceptionally dynamic during brain development and aging (Jin et al., 2011; Szulwach et al., 2011), suggesting that they play critical roles.

**FIGURE 5:**
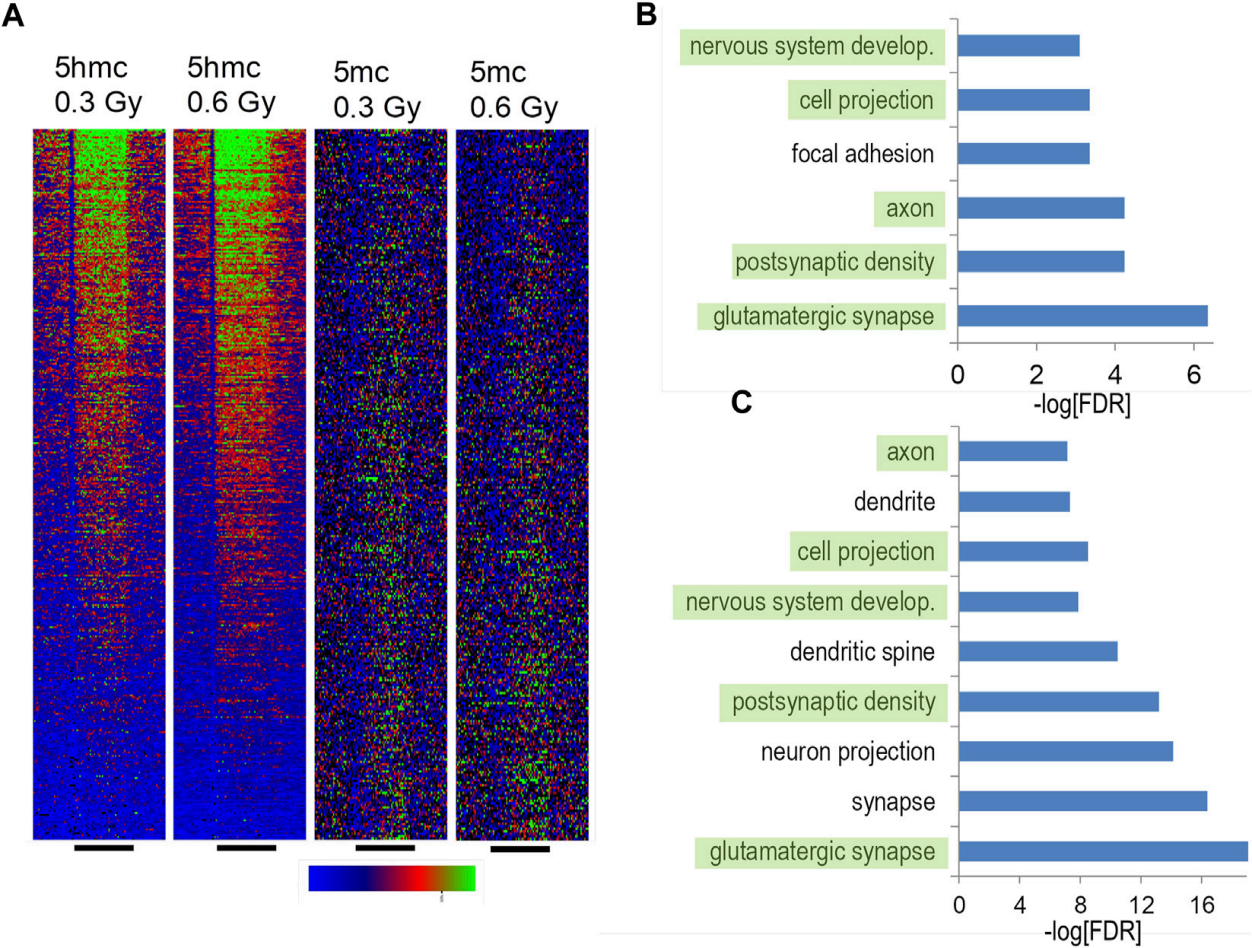
Hippocampal DNA methylation and hydroxymethylation pathways regulated by silicon irradiation. **(A)** DIP density histograms illustrating 5 mC and 5 hmC signals at RefSeq genes sorted by RNA-Seq gene expression levels. The antibodies used to pull down 5mC and 5 hmC regions do not cross react. **(B)** Gene ontology analyses of upregulated 5 hmC DHRs irradiated with 0.3 Gy. DHRs were annotated with the closest RefSeq gene start site within 50 kb. **(C)** Gene ontology analyses of upregulated 5 hmC DHRs irradiated with 0.1 ^56^Fe Gy and reanalyzed using the same pipeline as the silicon data. DHRs were annotated with the closest RefSeq gene start site within 50 kb.

Recently, we reported alterations in hippocampal DNA methylation following proton (Impey et al., 2016b; Impey et al., 2017) and ^56^Fe ion (Impey et al., 2016a) irradiation. To determine the effects of ^28^Si ion irradiation on hippocampal DNA methylation, we generated cytosine methylation (5 mC) and/or cytosine hydroxymethylation (5 hmC)-DIP-Seq libraries from the hippocampi of sham irradiated mice and mice exposed to 0.3 or 0.6 Gy of ^28^Si ion radiation using highly specific antibodies (Impey et al., 2016a; Impey et al., 2016b). These libraries were sequenced to an average depth of ∼14 million tags of which ∼61% mapped to a unique genomic location (Supplementary Table S1). Because 5mc-DIP-Seq tends to map to repetitive areas the lower percentage of unique alignments relative to 5 hmc-DIP-Seq is expected. Genomic regions enriched for 5 mC or 5 hmC were segmented using a previously published Monte Carlo-based algorithm (Impey et al., 2016a; Impey et al., 2017; Johnson et al., 2017) and the union of these regions was tested for significant differences based on a negative binomial distribution (Love et al., 2014). This pipeline identified thousands of differentially methylated and hydroxymethylated regions (DMRs and DHRs, respectively), of which the majority were within 25 kb of a transcription start site (Figure 5A).

Consistent with our and other labs’ DIP-Seq data, DMRs and DHRs distribution showed an expected bias towards genes with high levels of expression or low levels of expression respectively (Figure 5A). Also consistent with our analyses of ^56^Fe irradiation and literature indicating that 5 hmC is a stable epigenetic mark associated with gene expression in brain (Hahn et al., 2014), there was a highly significant enrichment for gene ontology annotation for increased DHRs at the 0.3 Gy dose (Figure 5B). There was weak enrichment for one gene ontology category (“glutamatergic synapse”, FDR-adjusted *p* < 5 × 10^−4^) for decreased DHRs at the 0.3 Gy while all other DHR or DMR conditions showed no significant enrichment for gene ontology categories. Therefore, in this study we focused further analyses on DHRs that showed a significant increase. There was a striking overlap between gene ontology pathways linked to synaptic function and development between the 0.3 Gy ^28^Si dose and our previous ^56^Fe 0.1 Gy data (Figures 5B, C) (Impey et al., 2016a), suggesting that these two forms of space radiation regulate similar DNA methylation responses. Consistent with this, there was only a ∼14% overlap between ^28^Si DHR-associated gene ontology categories and our previously-reported proton-irradiation DHR-associated gene ontology analysis (Impey et al., 2017).

### 3.4 Hippocampal radiation signature

**FIGURE 6:**
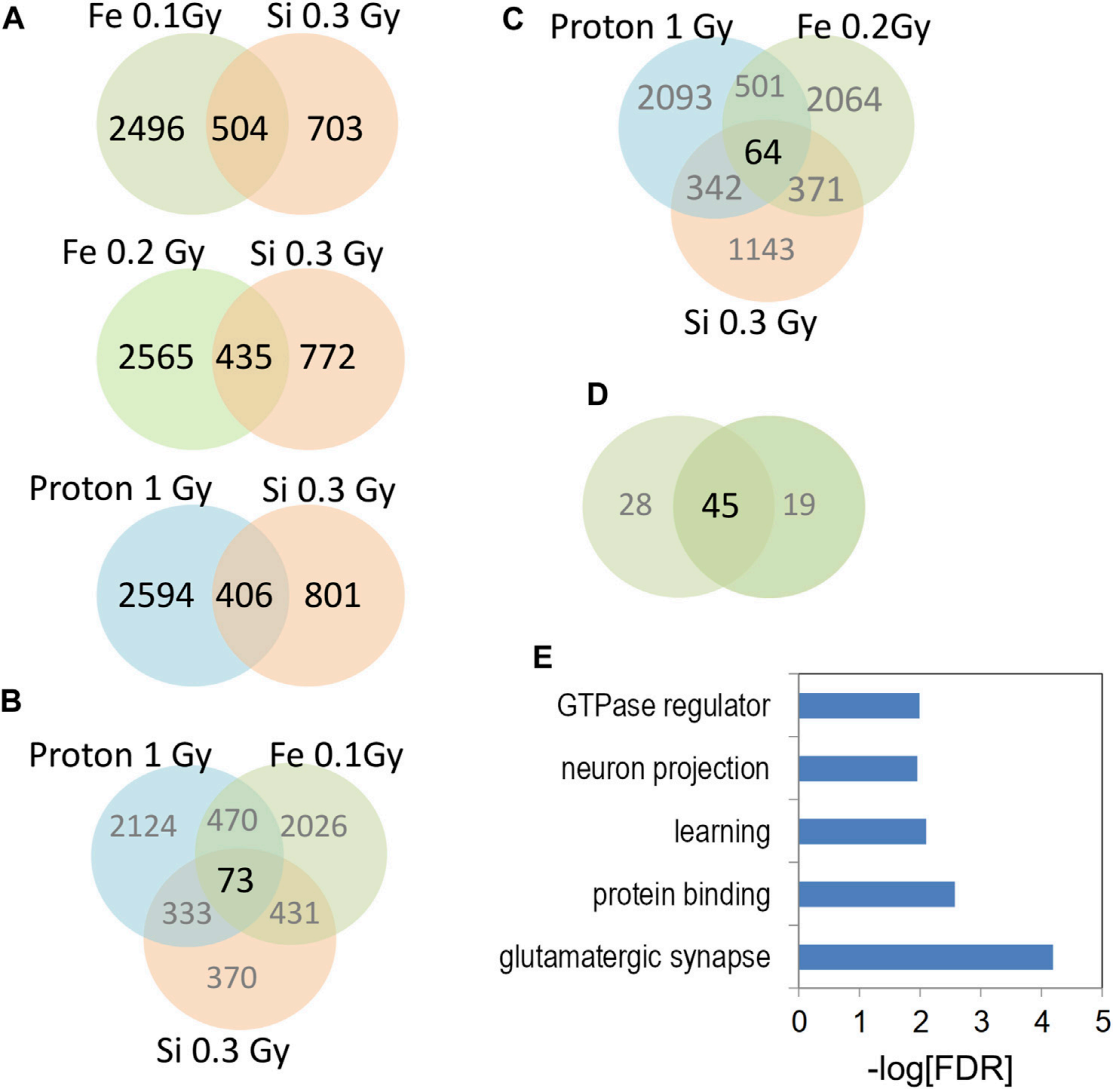
**(A)** Venn diagrams depict the overlap of gene-associated DHRs significantly increase by the indicated radiation treatment. DHRs were selected by *p*-value rank (top 1,500) and absolute tag difference (>10). DHRs were annotated with the closest RefSeq gene start site within 50 kb. **(B,C)** Venn diagrams depict the three way overlap of gene-associated DHRs significantly increased by the indicated radiation treatments. **(D)** Venn diagram illustrates the overlap between the 3-way intersections in panels **(B,C)**. **(E)** Gene-ontology analyses of 45 genes from **(D)**.

We next sought to compare 0.3 Gy ^28^Si DHRs (600 MeV/n) with previously published proton (150 MeV; 1 Gy) (Impey et al., 2016b) (Impey et al., 2016b), and ^56^Fe data (600 MeV/n, 0.1 Gy) (Impey et al., 2016a) by selecting the most-significant gene-associated DHRs from all data sets (top 3,000 DHRs ranked by *p*-value; *p* < 0.01 cut off; duplicate genes removed) and testing for overlap of resulting RefSeq genes. These comparisons revealed highly significant overlap between ^28^Si DHR-associated genes and analogous ^56^Fe and proton DHR-associated genes (Figure 6A). Interestingly, the overlap between the 0.1 Gy ^56^Fe and 0.3 Gy ^28^Si sets was markedly more significant (0.3 Gy ^28^Si vs. 0.1 Gy ^56^Fe, Fisher exact *p* < 2 × 10^−69^; 0.3 Gy ^28^Si vs. 0.2 Gy ^56^Fe, Fisher exact *p* < 4 × 10^−37^) than the overlap between the 0.3 Gy ^28^Si and proton 1 Gy data (Fisher exact *p* < 5 × 10^−48^) (Figure 6A). The overlapping set of DHRs led us to determine the subset of DHR-associated genes that were in common to all three forms of radiation exposure. We identified overlapping genes that were associated with increased hydroxymethylation in response to all three forms of radiation (Superexact test: Figure 6B, *p* < 3 × 10^−6^; Figure 6C *p* < 3 × 10^−4^). The intersection of all 4 data sets was very highly significant (Superexact test: Figure 6D *p* < 3 × 10^−20^) and this 45 DHR-associated gene “signature” was significantly enriched for “glutamatergic synapse”, “learning”, and “neuron projection” gene-ontology categories which suggest that this set of DHR-associated genes denotes a pathway linked to changes in synapse function and synaptic plasticity (Figure 6E).

**FIGURE 7:**
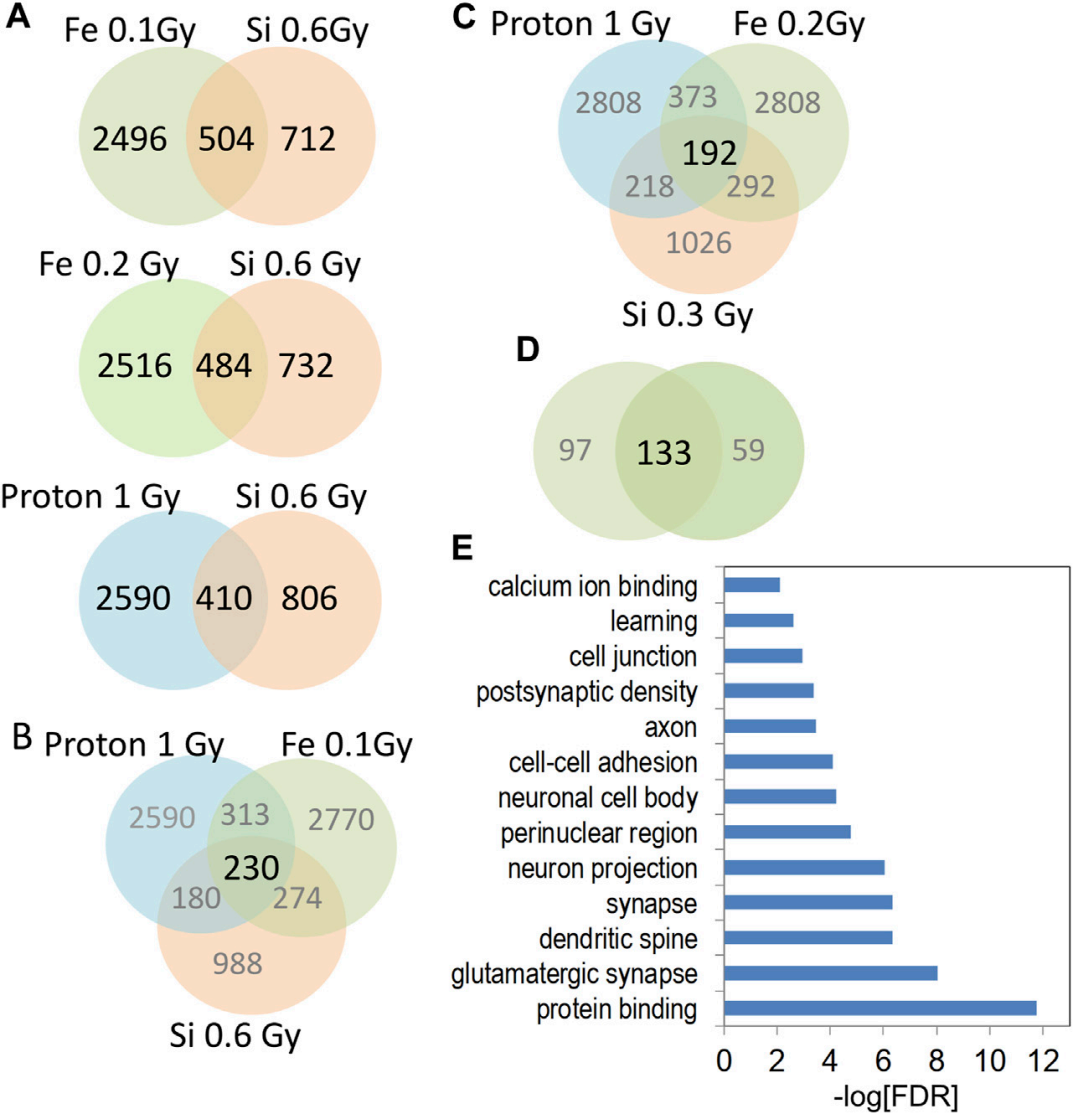
**(A)** Venn diagrams depict the overlap of gene-associated DHRs significantly increase by the indicated radiation treatment. DHRs were selected by *p*-value rank (top 1,500) and absolute tag difference (>10). DHRs were annotated with the closest RefSeq gene start site within 50 kb. **(B,C)** Venn diagrams depict the three way overlap of gene-associated DHRs significantly increased by the indicated radiation treatment. **(D)** Venn diagram illustrates the overlap between the 3-way intersections in panels **(B,C)**. **(E)** Gene-ontology analyses of 133 genes from **(D)**.

A similar set of analyses that compared 0.6 Gy ^28^Si DHR-associated genes with ^56^Fe and proton DHR-associated genes also found more significant overlap for the 0.1 Gy ^56^Fe data (Figure 7A: 0.6 Gy ^28^Si vs. 0.1 Gy ^56^Fe, Fisher exact *p* < 5 × 10^−68^) than for the proton 1Gy (Fisher exact, *p* < 9 × 10^49^) or 0.2 Gy ^56^Fe comparisons (0.6 Gy ^28^Si vs. 0.2 Gy ^56^Fe, Fisher exact *p* < 4 × 10^−30^). The DHR-associated gene overlap between all three forms of radiation was again highly significant (Figure 7B, *p* < 2x^−100^; Figure 7C, *p* < 7 × 10^−70^) as was the overlap between the proton, ^28^Si, and two doses of ^56^Fe radiation (Figure 7D, SuperExact test, 9 × 10^−200^).

**FIGURE 8:**
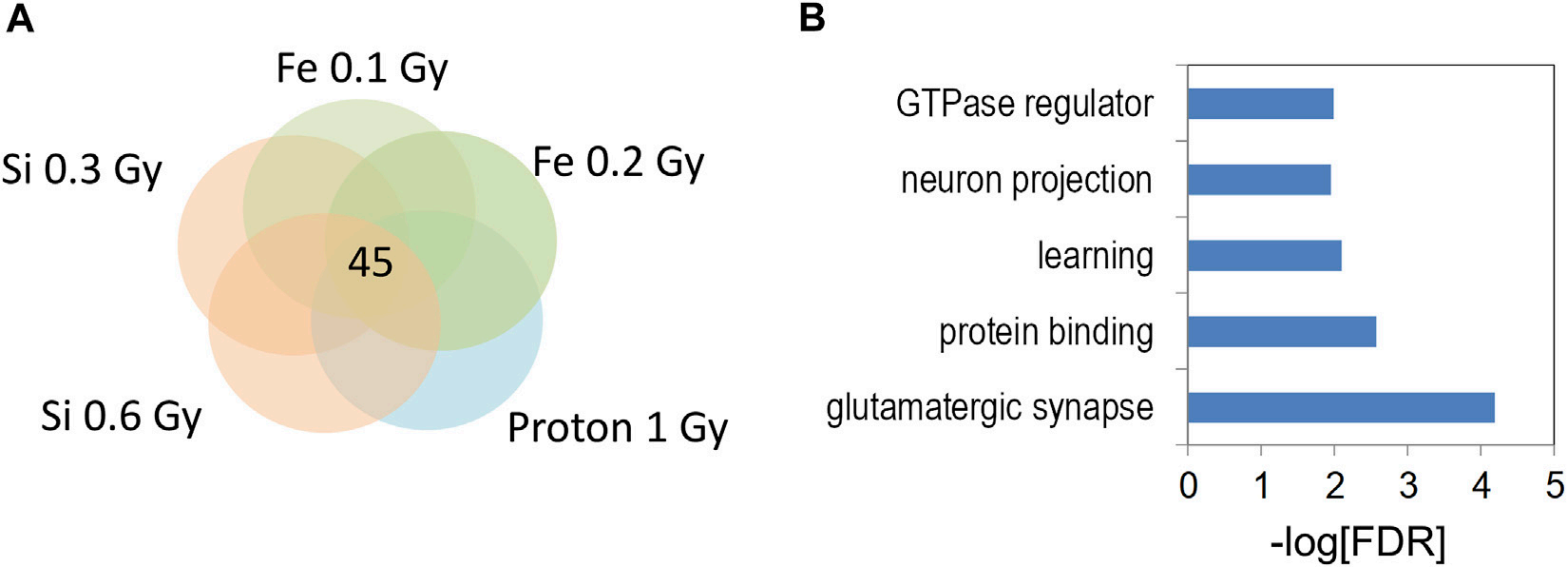
**(A)** Venn diagram illustrates the five way overlap of gene-associated DHRs significantly increased by indicated radiation treatments in Figures 5, 6. **(B)** Gene ontology analyses of 5 hmC DHRs from 5-way intersection in **(A)**.

The 133 gene “4-way” intersection (Figure 7D) was significantly enriched for gene ontology categories linked to synaptic function and neuron projection (Figure 7E) suggesting that 0.6 Gy ^28^Si irradiation induced a response more closely correlated with our published ^56^Fe and proton radiation responses (Impey et al., 2016a; Impey et al., 2016b). We next tested whether this 133 gene space radiation “signature” contained genes previously linked to radiation in rodent and human models (Xu et al., 2020) and found a non-significant intersection (Table 1) that included genes involved in growth-factor and small GTPase signaling (WIPF1, PIAS1, and IGFR1). Remarkably, the 45 genes in the 0.3 Gy ^28^Si “4-way” intersection (Figure 6D) were all present in the 133 gene 0.6 Gy ^28^Si “4-way” intersection (Figure 8A, SuperExact test, *p* < 7 × 10^−68^) indicating that there is a DHR-associated gene signature shared between the two ^28^Si doses (0.3 Gy, 0.6 Gy), the two ^56^Fe doses (0.1 Gy, 0.2 Gy), and the 1 Gy proton dose (Figure 8B; Table 2) (Cekanaviciute et al., 2023). This space radiation signature of 45 genes contained only one gene, PIAS1, previously linked to radiation response in a database largely limited to “photon” radiation studies (Xu et al., 2020). To provide better context for this space-radiation hydroxymethylation-related gene signature we annotated each gene using PubMed and Google Scholar searches for “radiation”, “ionizing radiation,” or “space radiation;” 23 of 45 genes were associated with studies indicating a radiation response ranging from “radiation response” gene ontology category membership (3), changes in gene expression (19), differential methylation of genetic loci (2), and radiation-associated mutagenesis or crosslinking (3) (Table 2). Interestingly, Npas3 was identified as a significant space-radiation decreased gene (^12^C ion beam) (Iwakawa et al., 2008) while Ncald and Parkn were identified as genes that are preferentially mutated in response to space radiation-associated DNA damage (^40^Ar and ^65^Fe ion beam) (Cekanaviciute et al., 2023).

**TABLE 1:** Intersection between 133 gene space radiation signature and Radatlas ionizing-radiation-regulated genes (MESH terms).

Gene symbol	Gene name
Cyfip2	Cytoplasmice FMR1 interacting protein
Etv6	Ets variant 6
Grm1	Glutamate receptor, metabotropic 1
Igf1r	Insulin-like growth factor I receptor
Pias1	Protein inhibitor of activated STAT1
Usp15	Ubiquitin specific peptide
Wipf1	WAS/WASL interacting protein family, member 1

**TABLE 2:** Summary of the novel object recognition data of the ^56^Fe ion (600 MeV) and proton (150 MeV) studies^a^.

Study, doses, and energies	Dose impairing cognitive performance (Gy)	Dose not impairing cognitive performance
^56^Fe (600 MeV): 0.1, 0.2, and 0.4 Gy	0.1	0.2 and 0.4 Gy
28Si (600 MeV): 0.3, 0.6, and 0.9 Gy	0.3	0.6 and 0.9 Gy
Protons (150 MeV): 1 Gy	1	

^a^ The mice in these three studies were all purchased as 6-month-old C57BL/6J wild-type male mice from JAX, irradiated at BNL, and tested at OHSU. The time line of testing and euthanasia matches that of the current study.

### 3.5 RNA-Seq

**FIGURE 9:**
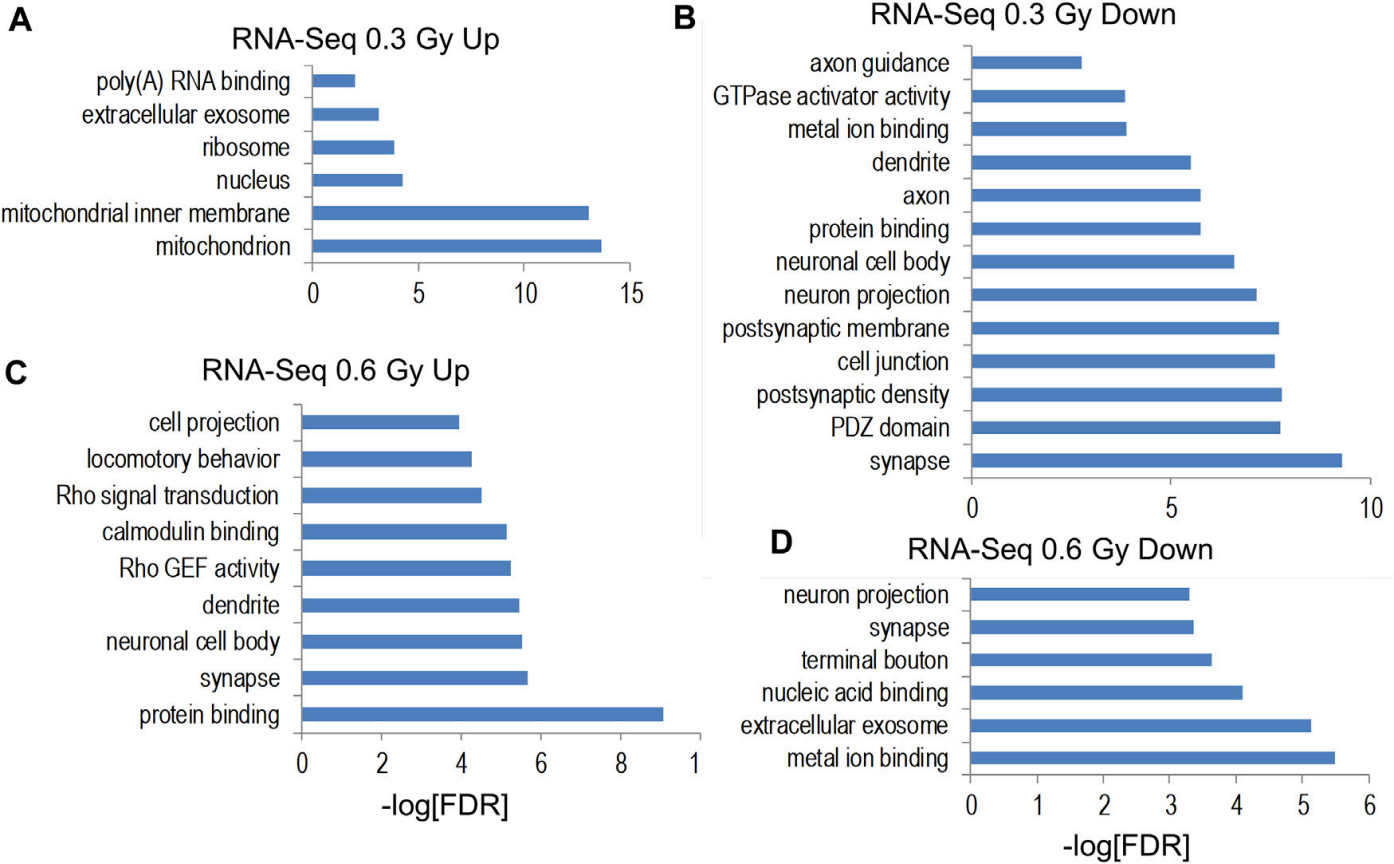
**(A–D)** Bar graphs depicts gene ontology analyses of RefSeq genes significantly up- and downregulated (*p* < 0.01) for indicated RNA-Seq comparisons.

We next utilized RNA-Seq to profile the transcriptional response to ^28^Si irradiation using hippocampal RNA co-extracted from the same tissue used for DIP-Seq experiments. Although our ^56^Fe irradiation study (Impey et al., 2016a) identified highly significant changes in gene expression that correlated with DHRs (but not DMRs), we did not observe a significant transcriptional response to either 0.3 Gy or 0.6 Gy ^28^Si irradiation at the 2 weeks time point (no genes reached the FDR-adjusted *p* < 0.01 cut off, Supplementary Tables S1, S2). This result is consistent with our observation that ^28^Si irradiation at the 2 weeks time point only demonstrated significant gene ontologies at the 0.3 Gy dose whereas we saw highly significant gene ontology and KEGG pathway enrichment for both down and upregulated DHRs following 0.1 Gy and 0.2 Gy ^56^Fe irradiation (Impey et al., 2016a). Because gene expression pathway data is often regulated in a biologically meaningful manner even when individual genes do not achieve FDR-adjusted significance, we selected differentially-expressed genes at an unadjusted *p*-value cut off of *p* < 0.01 and assessed gene-ontology category and KEGG pathway enrichment via the Fisher-exact test. For genes increased by the 0.3 Gy ^28^Si dose, gene ontology categories associated with mitochondrial oxidative phosphorylation were most-significantly enriched as was the KEGG pathway for oxidative phosphorylation (Figure 9A; Supplementary Figure S1). Interestingly, we also observed significant enrichment for KEGG pathways associated with Huntington’s, Alzheimer’s, and Parkinson’s diseases (Supplementary Figures S2–S4). We also saw enrichment for Huntington’s, Alzheimer’s, and Parkinson’s disease KEGG pathways for genes significantly decreased by ^56^Fe irradiation in a previous study (Impey et al., 2016a), suggesting that these pathways may represent a more generalized space-radiation response. Because oxidative stress has been linked to the neuropathology of these neurodegenerative disorders (Kim et al., 2015), our data suggest that a 0.3 Gy ^28^Si radiation dose may trigger persistent oxidative stress that adversely impacts neuronal homeostasis/metabolism. Genes decreased by 0.3 Gy ^28^Si irradiation were significantly enriched for synaptic components and neuronal proteins (Figure 9B) which is consistent with the idea that the radiation-induced oxidative stress adversely impacts synaptic function/homeostasis.

**FIGURE 10:**
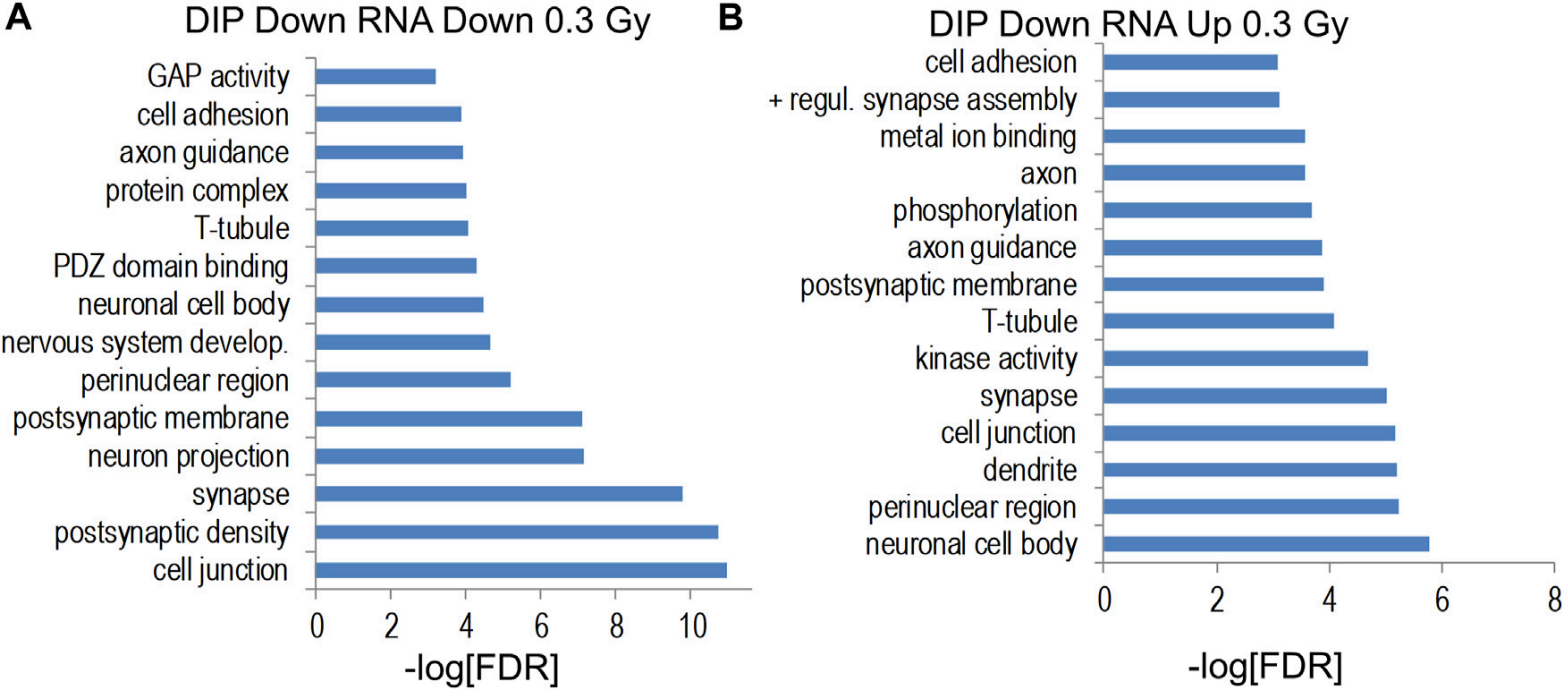
**(A,B)** Gene ontology analyses of indicated intersections between gene-annotated DHRs and differentially-expressed genes (unadjusted *p* < 0.011).

Genes increased by 0.6 Gy ^28^Si irradiation were enriched for gene ontology categories associated with synaptic and neuronal proteins (Figure 9C) while genes decreased by 0.6 Gy ^28^Si irradiation showed less-significant enrichment for neuron-associated gene ontology categories (Figure 9D) and KEGG pathways (Supplementary Figure S5). The Alzheimer’s disease KEGG pathway was significantly enriched for genes decreased by 0.6 Gy ^28^Si irradiation which is the opposite effect seen for 0.3 Gy ^28^Si. It is conceivable that the 0.6 Gy ^28^Si dose triggered compensatory neuroprotective gene expression changes. Gene ontology analyses of indicated intersections between gene-annotated DHRs and differentially-expressed genes are illustrated in Figure 10.

### 3.6 Relationship between DNA hydroxymethylation and gene expression

We next assessed the correlation between gene-associated differential DNA hydroxymethylation and gene expression. Although we did not detect the highly-correlated spatial correlation of DHRs and gene expression seen for ^56^Fe irradiation (Impey et al., 2016a), we found significant overlap between downregulated genes and both increased and decreased DHRs at the 0.3 Gy and 0.6 Gy ^28^Si radiation doses (Supplementary Figure S6). These data suggest that by intersecting DIP-Seq data with RNA-Seq data, we increased statistical power allowing us to detect biologically relevant epigenetic remodeling. Consistent with this idea, the highly significant intersection between decreased 0.3 Gy ^28^Si DHRs and both up and downregulated genes were significantly enriched for gene ontology categories linked to synapses, neuronal connectivity, and neuronal development (Supplementary Figure S7). We also identified the “mitochondrion” gene ontology category when comparing increased 0.3 Gy ^28^Si DHRs with increased 0.3 Gy ^28^Si gene expression and the “nervous system development” gene ontology category for the intersection of decreased 0.6 Gy ^28^Si DHRs with decreased 0.6 Gy ^28^Si gene expression.

## 4 Discussion

The results of the current study show that at 0.3 Gy, but not at 0.6 Gy or 0.9 Gy, ^28^Si ion irradiation impaired cognition and network activity in the CA3 region of the hippocampus. Consistent with this pattern of the dose-response curve for cognitive performance, sham-irradiated mice and those irradiated with ^56^Fe (600 MeV) at 0.2 Gy showed novel object recognition but mice irradiated with 0.1 Gy were impaired (Impey et al., 2016a) (Table 2 for Summary of the cognitive data of the ^56^Fe (600 MeV) and proton (150 MeV) studies). This pattern is especially concerning as the lower doses would be expected to be more pertinent to doses astronauts will be exposed to during space missions. While the percentage of *Arc*-positive neurons expressing *Arc* mRNA in the nucleus and cytoplasm in the CA3 region of the hippocampus of sham-irradiated mice was higher following exposure twice to the same environment, as opposed to exposure to two different environments, this was not seen in mice irradiated with 0.3 Gy that showed impaired cognitive performance. In mice irradiated with 0.9 Gy, who showed no impaired cognitive performance, there was a trend towards a difference in the CA3 but not the CA1 region. The CA3 region is involved in rapid encoding of new detailed memories and bound to a large degree to a spatial coordinate system and more important for distinguishing different environments, while the downstream CA1 region can interpret this information from the CA3 region in the context of less spatially restricted information and is more important for recognizing the same environment (Leutgeb et al., 2004; Leutgeb and Leutgeb, 2007). These data suggest that in mice irradiated with 0.3 Gy, the CA3 region might be especially affected, while in mice irradiated with 0.9 Gy, the CA3 region might be less affected than the CA1 region and sufficient for detecting a novel object in the environment. We detected significant enrichment of gene ontology categories only for DHRs at the 0.3 Gy ^28^Si dose but no significant enrichment of gene ontology categories for DMRs. As we observed following ^65^Fe and proton irradiation, DHRs were significantly associated with gene ontology pathways for synapses, postsynaptic specialization, and axons. In previous studies, when we detected gene-ontology category enrichment, it was consistently more significant for 5 hmC data than for 5 mC data. Moreover, for multiple conditions (^65^Fe 20 weeks, proton radiation 2 weeks), there was a significant gene ontology enrichment only for DHRs. Therefore, our results are consistent with our general observation that space-radiation-associated 5 hmC remodeling is more tightly associated with genes and gene pathways than 5 mC remodeling. It should be noted that because 5 hmC is generated by enzymatic hydroxylation of 5mC, remodeling of 5 mC is also captured by methodologies that measure changes in 5 hmc. Interestingly, the 0.3 Gy ^28^Si dose was also the only dose that caused a significant deficit in novel object recognition learning, suggesting that 5 hmC remodeling of synapse-associated gene ontology category may be linked to cognitive dysfunction. These data show that ^28^Si ion radiation-induced cognitive injury is associated with hippocampal changes in DNA hydroxymethylation of cytosine, a major epigenetic modification involving dynamic changes methyl groups, and specific gene ontology categories linked to synaptic pathways and the post-synaptic density. Consistent with these data, hippocampal changes in DNA methylation are involved in the regulation of expression of genes required for cognitive performance (Miller and Sweatt, 2007; Lubin et al., 2008). 5 hmC DNA methylation is derived from 5 mC by the action of three TET enzymes (TET 1–3), is hydroxymethylcytosine (5 hmC) (Veron and Peters, 2011). While in brain, levels of TET2 are higher than those of TET1 or TET3 in the brain, and therefore TET2 is believed most important for brain function (Chen et al., 2012; Dzitoyeva et al., 2012); however, we did not see effects of radiation on TET2 levels.

While altered hippocampal DNA methylation was seen following 0.6 Gy 28Si ion irradiation, there was no impairment in novel object recognition. Genes increased by 0.6 Gy ^28^Si irradiation were enriched for gene ontology categories associated with synaptic and neuronal proteins. In addition, the Alzheimer’s disease KEGG pathway was significantly enriched for genes decreased by 0.6 Gy, but increased by 0.3 Gy ^28^Si irradiation. Based on these results, we hypothesize that the 0.6 Gy ^28^Si dose triggered compensatory neuroprotective gene expression changes that prevented impairments in novel object recognition seen following 0.3 Gy.

We next assessed whether there is a general space radiation synaptic signature and/or distinct changes in hippocampal DNA methylation comparing effects of proton, ^56^Fe ion, and ^28^Si ion irradiation. Analyzing the shared hippocampal pathways affected 2 weeks following proton (150 MeV; 1 Gy), ^56^Fe (600 MeV/n, 0.1 Gy and 0.2 Gy) and ^28^Si (600 MeV/n, 0.3 Gy and 0.6 Gy) ion irradiation revealed 45 genes (Table 2). Although this set should not be taken as a total number of radiation response genes in the hippocampus because we only included those that were observed in five independent experiments with three different types of particles, their reproducible presence in these experiments represents a remarkable degree of specificity, and thus importance. This set of 45 genes was significantly enriched for gene ontology categories including “glutamatergic synapse”, “learning”, and “neuron projection”. The “glutamatergic synapse” gene ontology category included genes such as Neuron-glial related cell adhesion molecule (Nrcam), Parkin 2 (Prkn), Amphiphysin (Amph), Contactin associated protein-like 2 (Cntnap2)¸ and Sortilin-Related Receptor3 (Sorcs3). Interestingly, Nrcam, Amph, Cntnap2, and Sorcs3 regulate presynaptic glutamatergic function suggesting remodeling of genes involved in glutamate release. The “learning” gene ontology category was comprised of an overlapping set of enriched genes including Parkin 2 (Prkn), Amphiphysin (Amph), Contactin associated protein-like 2(Cntnap2)¸ Sortilin-Related Receptor3 (Sorcs3), and phospholipase C, beta 1 (Plcb1). The biological relevance of these associations is supported by a robust literature linking space radiation to changes in glutamatergic synapse gene expression, glutamatergic synapse function, and regulation of glutamatergic postsynaptic density genes/proteins (Machida et al., 2010; Allen et al., 2015; Parihar et al., 2015; Sokolova et al., 2015; Parihar et al., 2016; Krukowski et al., 2018; Parihar et al., 2018). Our results indicate that, in addition, to a microglial stress response (Parihar et al., 2016; Krukowski et al., 2018; Parihar et al., 2018) space radiation targets long-lasting gene expression and epigenetic remodeling responses to glutamatergic synapse genes.

Table 3 annotates these 45 genes by their association with radiation response in the scientific literature as well as their association with neurological disorders. The majority of these genes were associated with a radiation response and two (Asic2 and Celf4) were associated with both radiation-induced differential methylation and gene expression. Although only three genes (Asic2, Cntnap, and Pias1) were annotated in the “radiation response” gene ontology category another 20 were identified as radiation-regulated genes in the literature. Notably, one gene (Npas3) was identified as being decreased by space radiation (Iwakawa et al., 2008) and two genes (Nfia and Prkn) were identified as prone to space-radiation-mediated DNA damage (Cekanaviciute et al., 2023). The high degree of overlap between our 45 gene space radiation signature and the previous literature linking these genes to potential radiation response supports the idea that these sites of 5 hmC remodeling are biologically relevant to radiation injury The profound phenotype seen with those genes confirms the potential of this approach to identify genes and pathways involved in the CNS radiation response that are critical for CNS function.

**TABLE 3:** Comparing hippocampal DNA hydroxymethylation following proton, ^56^Fe ion, and ^28^Si ion irradiation revealed a general space radiation synaptic signature with 45 genes that are associated with profound phenotypes.

Gene symbol	Gene name	Radiation-response	Neurological disease
1700025G04Rik	RIKEN cDNA 1700025G04		
Shtn1	Shootin 1		
Asic2	acid-sensing (proton-gated) ion channel 1	GO: “Response to radiation” (Huang et al. (2011), Chiba et al. (2020); Radiation-regulated methylation	
Amph	amphiphysin		
Apba1	amyloid beta precursor protein binding, A 1		Linked to Alzheimer’s Disease
Asap1	ArfGAP with SH3 ankyrin repeat and PH domain 1		
Atxn7l1	ataxin 7-like 1		
Cdh4	cadherin 4		
Celf4	CUGBP, Elav-like family member 4	Radiation-induced Sallam et al. (2022); Radiation-regulated methylation Sallam et al. (2022)	
Chn2	chimerin 2		
Cntnap2	contactin associated protein-like 2	GO:“Response to radiation” Wang et al. (2020)	Mutation linked to Autism
Cux1	cut-like homeobox 1	Resistance to ionizing radiation Ramdzan et al. (2017); Radiation-induced DNA repair Vadnais et al. (2012)	
Dlg2	discs, large homolog 2	Radiation biomarker Rouchka et al. (2019)	Mutation linked to autism and schizophrenia
Dock1	dedicator of cytokinesis 1		
Dync1h1	dynein cytoplasmic 1 heavy chain 1		Mutation linked to autism and AD
Foxp1	forkhead box P1	Radiation-decreased Chiba et al. (2020)	Mutation linked to autism
Gfod1	glucose-fructose oxidoreductase domain 1		
Hs6st3	heparan sulfate 6-O-sulfotransferase 3	Radiation-induced Fachin et al. (2009)	
Itga9	integrin alpha 9		
Itsn1	intersectin 1	Radiation-decreased Michna et al. (2016)	
Klhl32	kelch-like 32		
Mcc	mutated in colorectal cancers	Radiation-phosphorylated Pangon et al. (2010)	
Ncald	neurocalcin delta	Persistently-induced by space radiation Cekanaviciute et al. (2023); Radiation-induced Klising-Sireul et al. (2006)	
Nfia	nuclear factor I/A	Radiation resistance regulator Sun et al. (2019); Radiation biomarker Biolatti et al. (2021)	
Nin	ninein	Radiation crosslinks to DNA Barker et al. (2005)	
Npas3	neuronal PAS domain protein 3	Space radiation-decreased Iwakawa et al. (2008)	Mutation linked Schizophrenia
Nrcam	neuronal cell adhesion molecule	Radiation-induced Godoy et al. (2013)	Mutation linked to Autism
Pard3b	par-3 family cell polarity regulator beta	Radiation-induced DNA repair Lees-Miller (2007)	Mutation linked Schizophrenia
Prkn	Parkinson disease 2	Persistently-induced by space radiation Cekanaviciute et al. (2023)	Mutation linked to Parkinson Disease
Pias1	protein inhibitor of activated STAT 1	GO: “Response to radiation” Fachin et al. (2009); Radiation-induced DNA repair Galanty et al. (2009)	
Plcb1	phospholipase C, beta 1	Radiation-increased Forrester et al. (2014)	Mutation linked to Epilepsy
Plce1	phospholipase C, epsilon 1		
Prex1	PtdIns(3,4,5)P3-dependent Rac exchange factor 1		Mutation linked to Autism
Ptprg	protein tyrosine phosphatase, receptor type, G	Radiation-decreased Yang et al. (2013)	
Rapgef1	Rap guanine nucleotide exchange factor 1	Radiation-induced Abend et al. (2015), Lee et al. (2014)	
Rbms1	RNA binding single stranded interacting protein 1		
Rfx4	regulatory factor X, 4	Radiation-decreased Loeliger et al. (2020)	Mutation linked to Autism and ADHD
Ryr2	ryanodine receptor 2, cardiac	Radiation-increased Mages et al. (2022); Radiation-induced phosphorylation Sag et al. (2013)	Mutation linked to epilepsy
Scfd2	Sec1 family domain containing 2		
Sorcs3	sortilin-related VPS10 domain receptor 3		Mutation linked to Schizophrenia
Stox2	storkhead box 2		
Syne1	spectrin repeat containing, nuclear envelope 1		
Tle1	transducin-like enhancer of split 1		
Wasf3	WAS protein family, member 3	Radiation-increased Kim et al. (2010)	
Xpr1	xenotropic and polytropic retrovirus receptor 1		

In summary, 0.3 Gy of ^28^Si ion irradiation (600 MeV/n) affects hippocampus-dependent cognitive performance, network activity in the CA3 region of the hippocampus, with impaired cognition correlating with altered gene expression and 5 hmC profiles that mapped to specific gene ontology pathways. The general space radiation synaptic signature with 45 genes that are associated with profound phenotypes following proton, ^56^Fe ion, and ^28^Si ion irradiation revealed that the most significant categories are glutamatergic synapse and postsynaptic density. Thus, the brain’s response to space irradiation involves novel excitatory synapse and postsynaptic remodeling. Future efforts are warranted to determine how this general radiation signature might be targeted to reduce detrimental effects of space radiation on the brain during and following missions.

## Data Availability

The datasets presented in this study can be found in online repositories. The names of the repository/repositories and accession number(s) can be found in the article/Supplementary Material.
