# Supplementary figures and images for: Postsynaptic density radiation signature following space irradiation
Source: Front Physiol. 2023 Jun 27;14:1215535. doi: 10.3389/fphys.2023.1215535 (PMC10334289; doi:10.3389/fphys.2023.1215535)

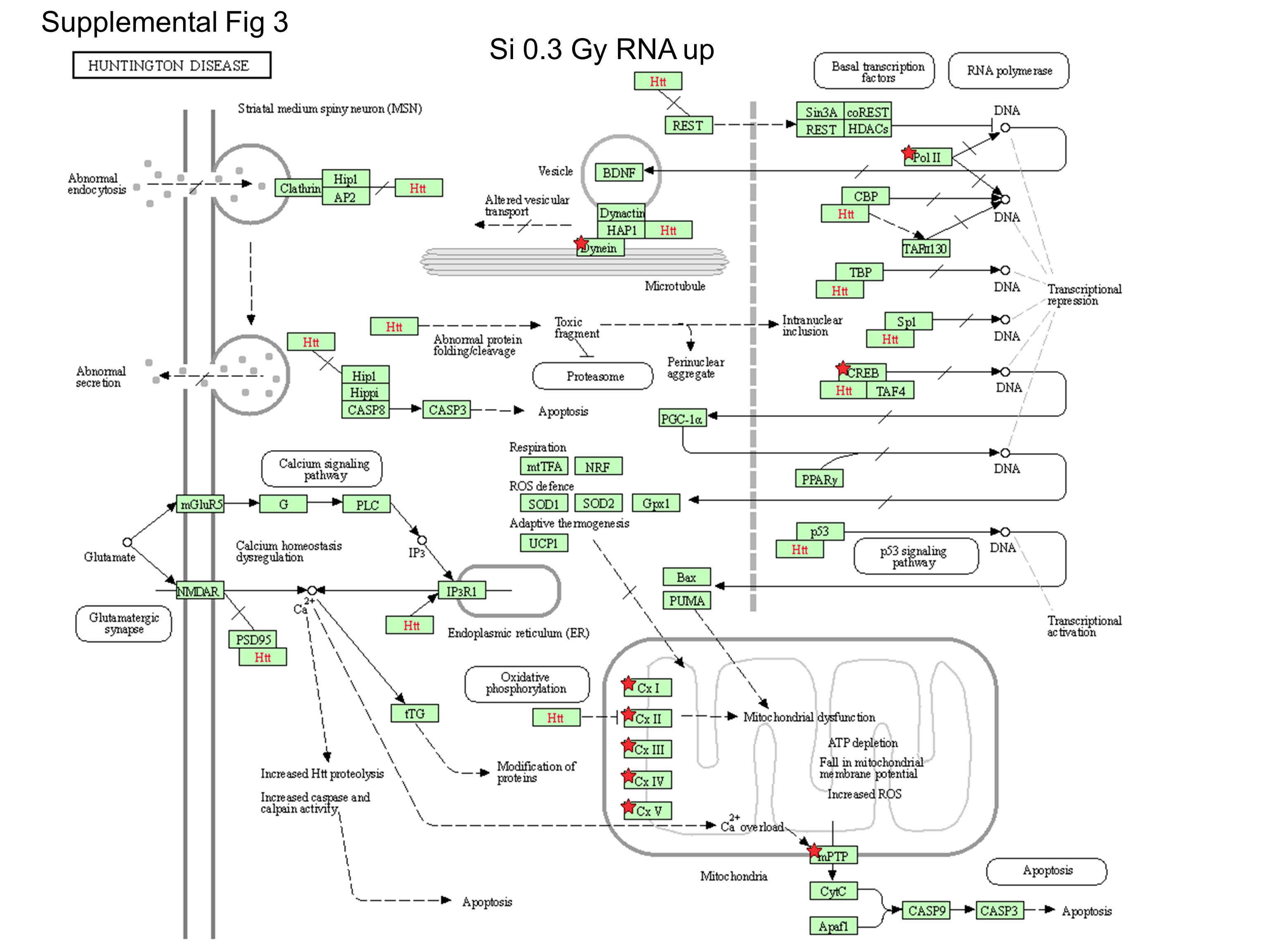

Supplement: Supplementary file 1 [file Image3.TIFF]

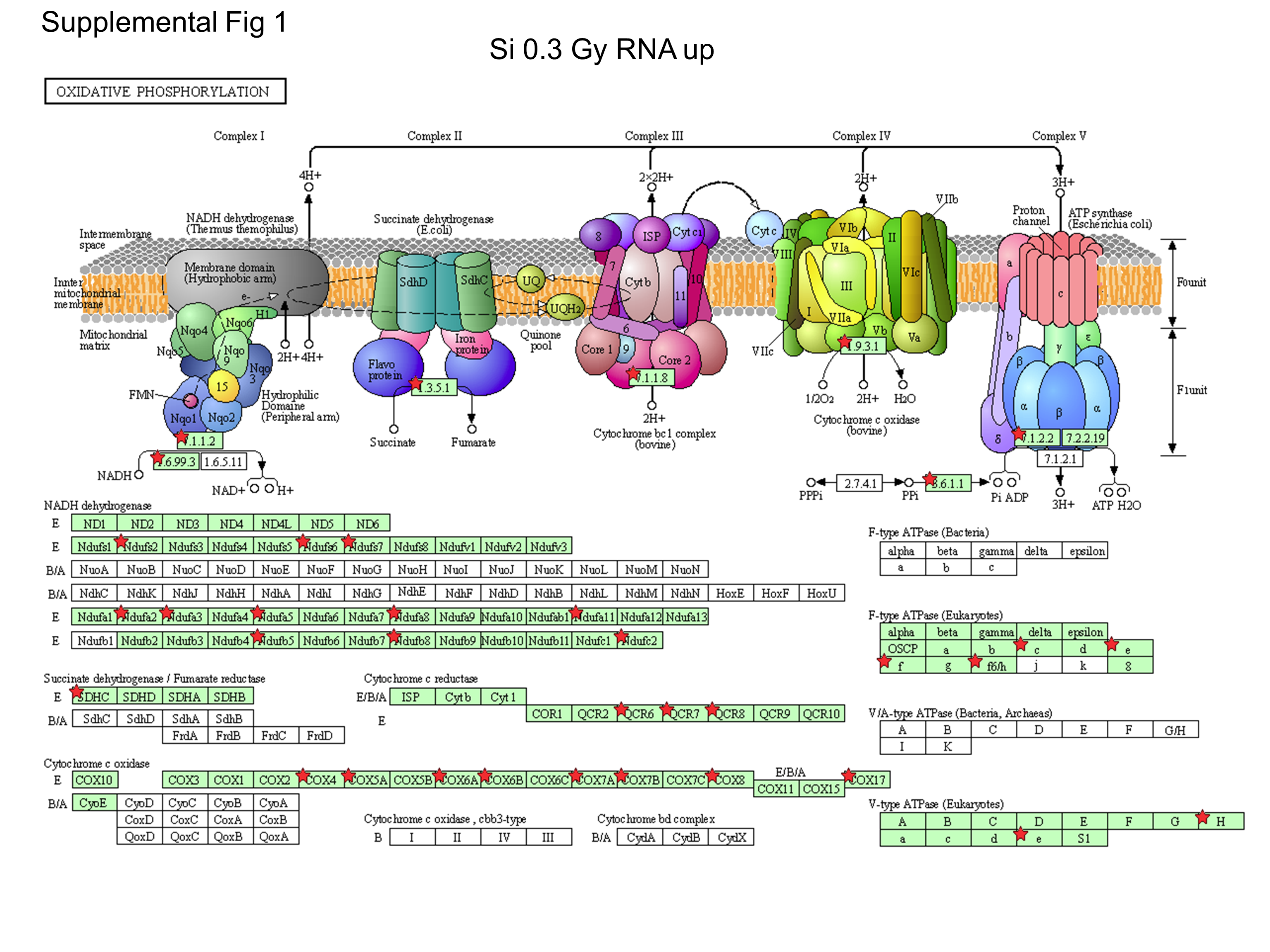

Supplement: Supplementary file 3 [file Image1.TIFF]

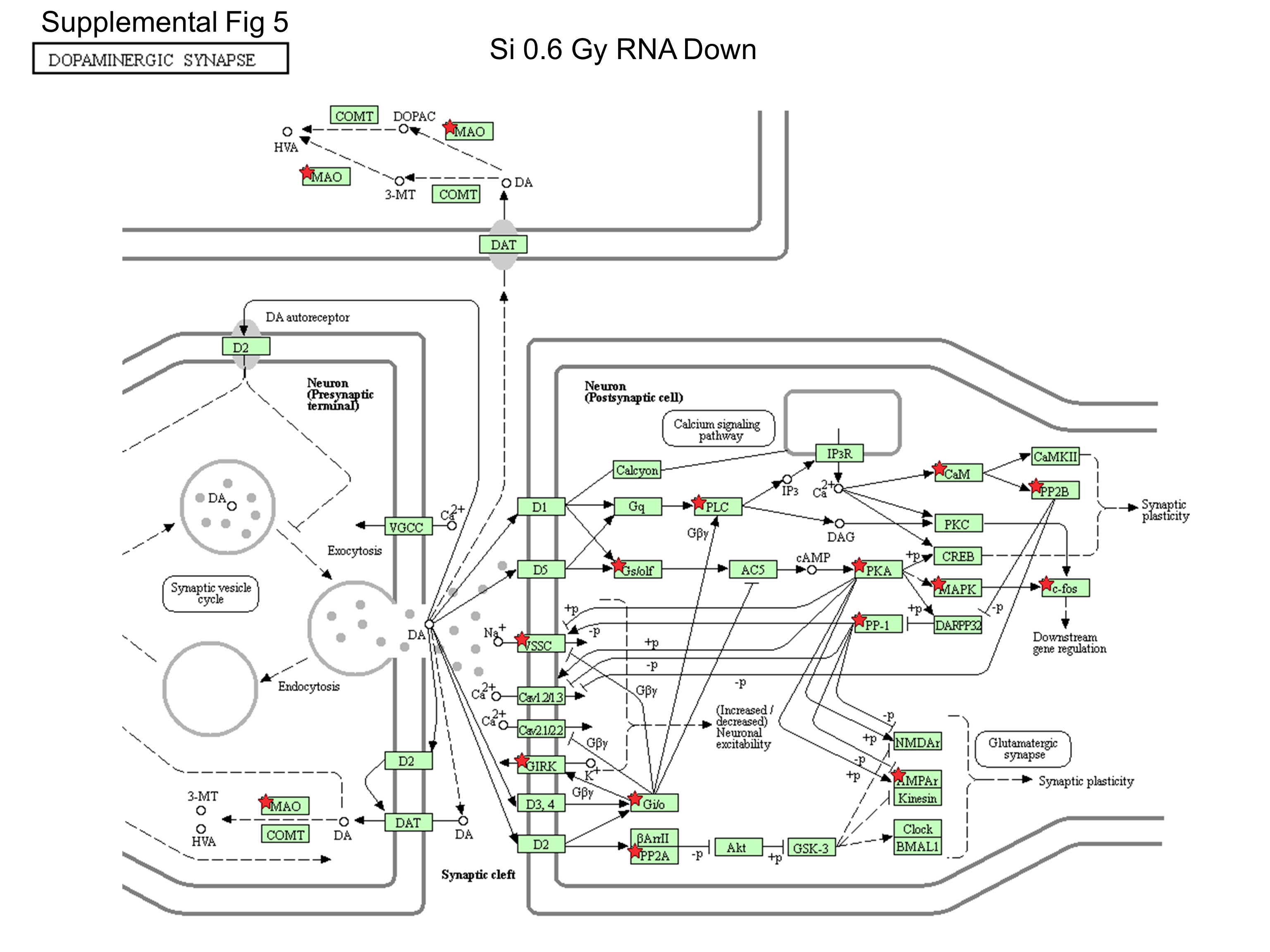

Supplement: Supplementary file 4 [file Image5.TIFF]

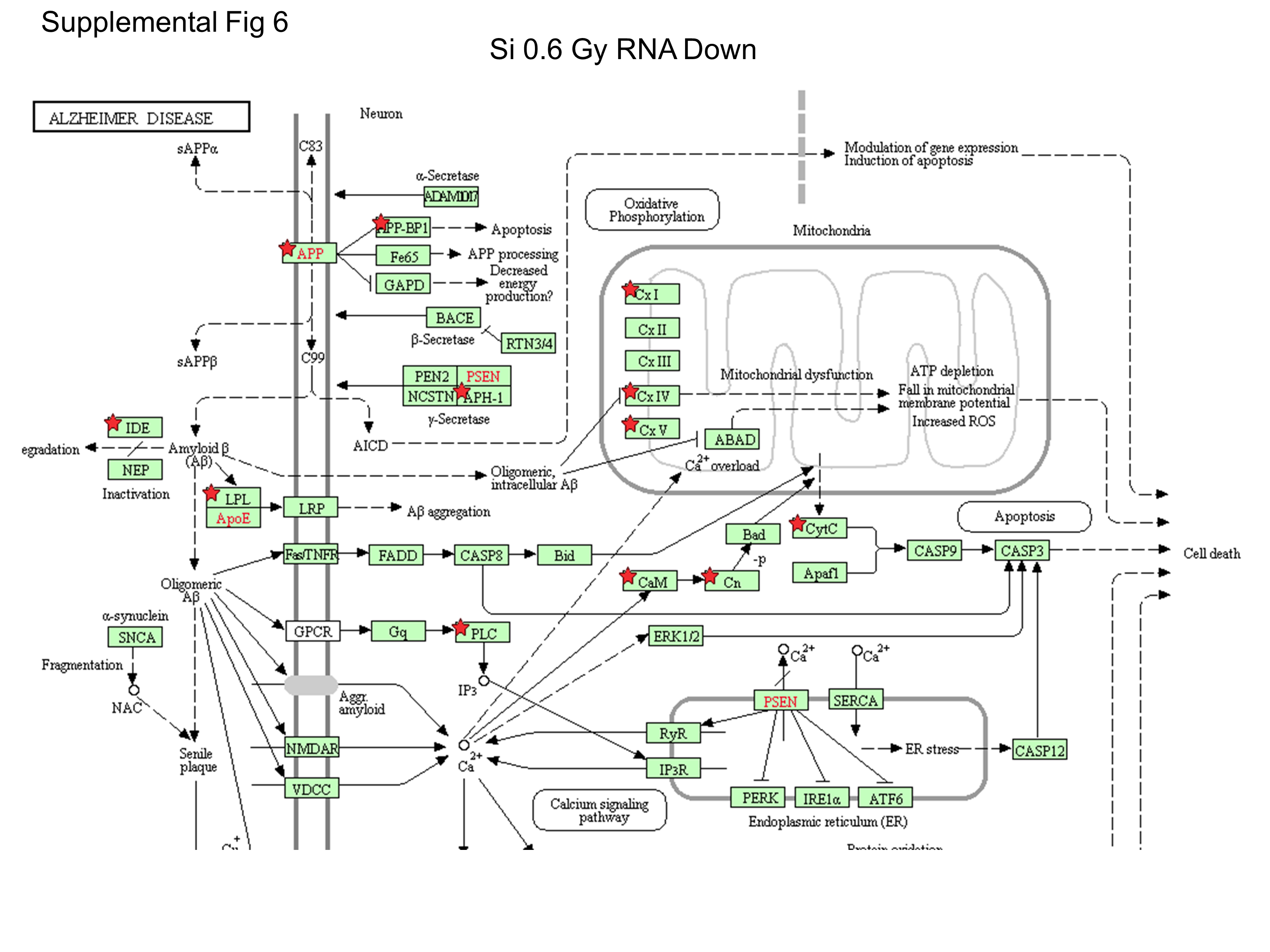

Supplement: Supplementary file 6 [file Image6.TIFF]

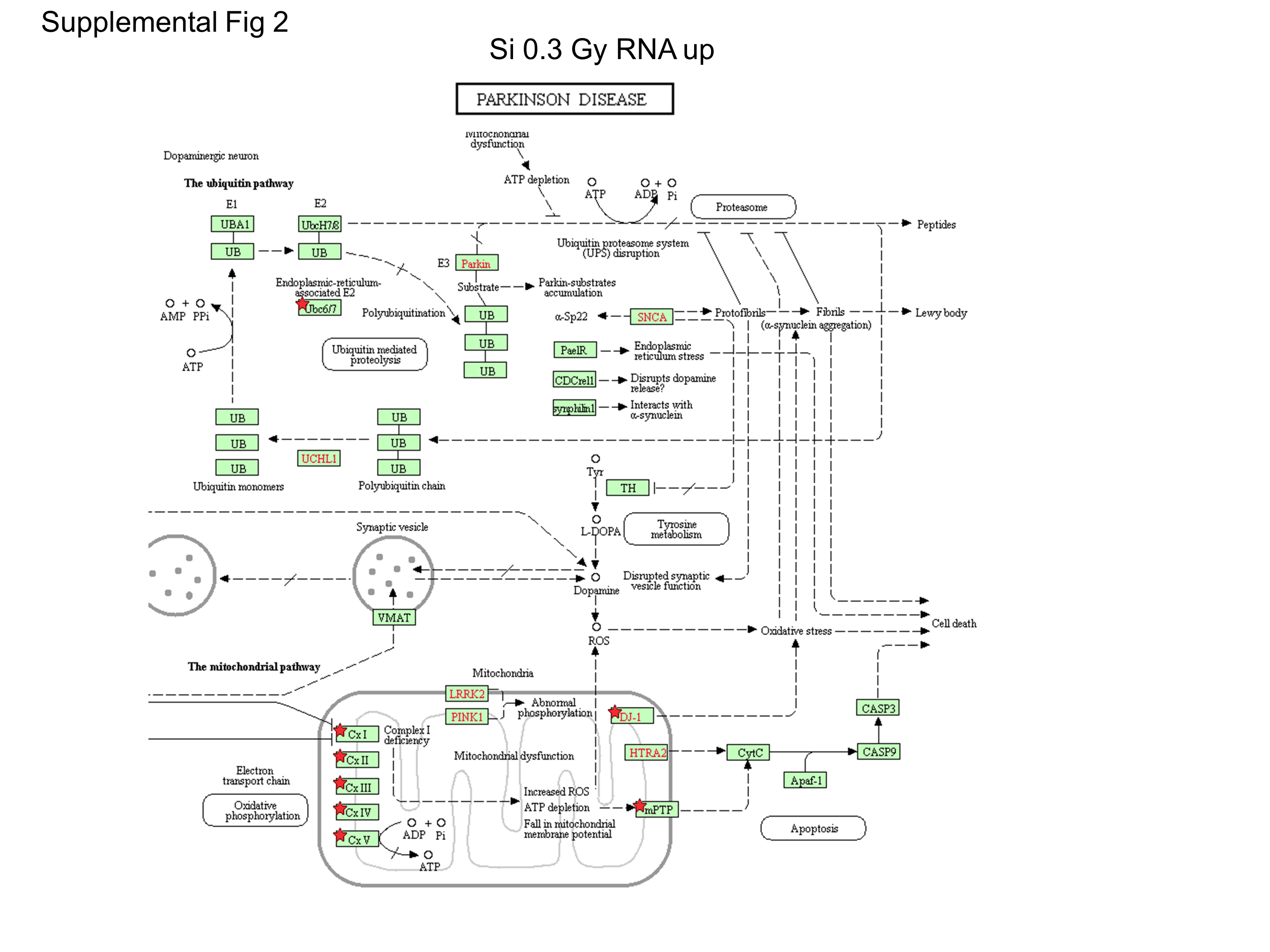

Supplement: Supplementary file 7 [file Image2.TIFF]

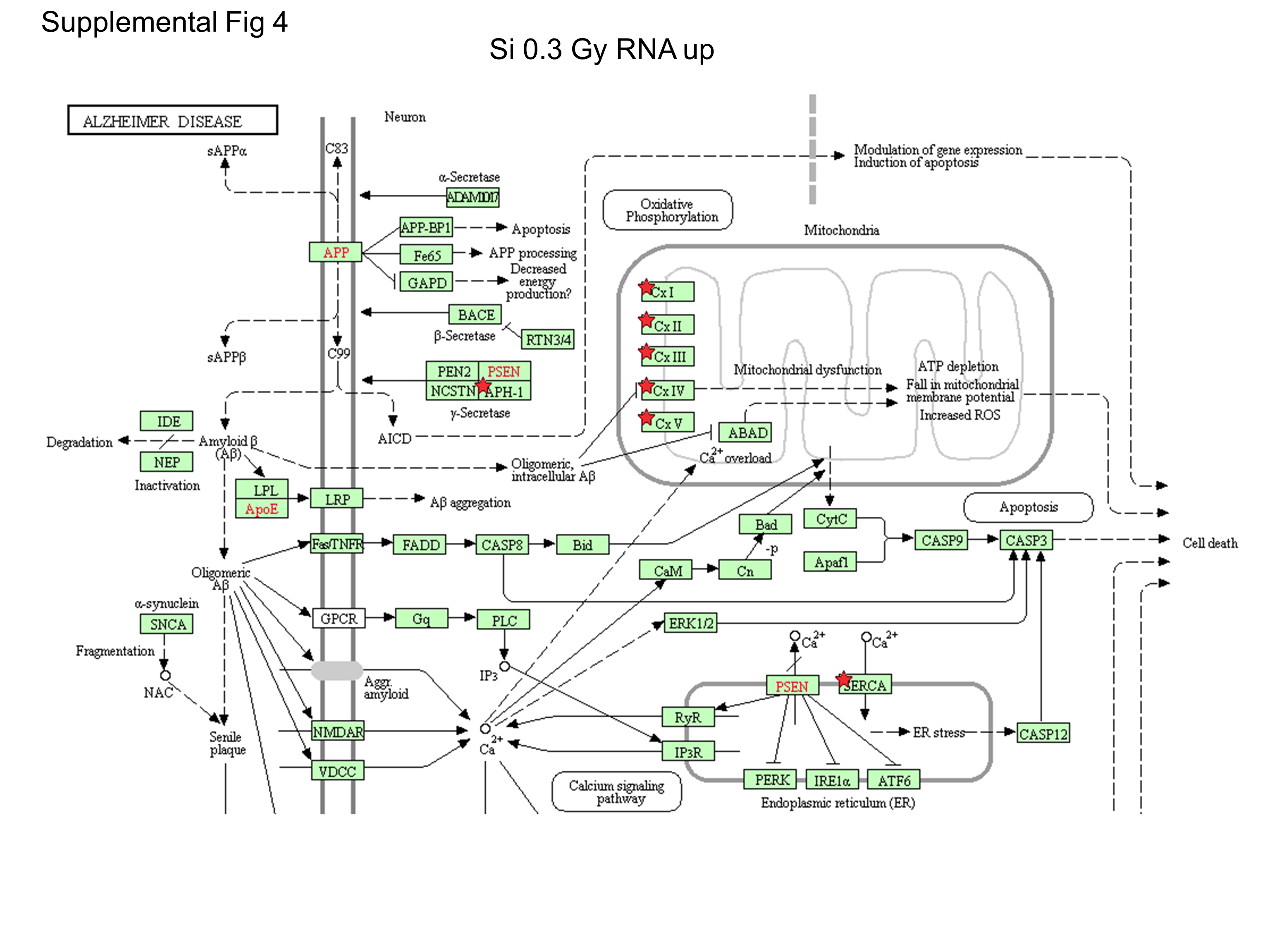

Supplement: Supplementary file 8 [file Image4.TIFF]

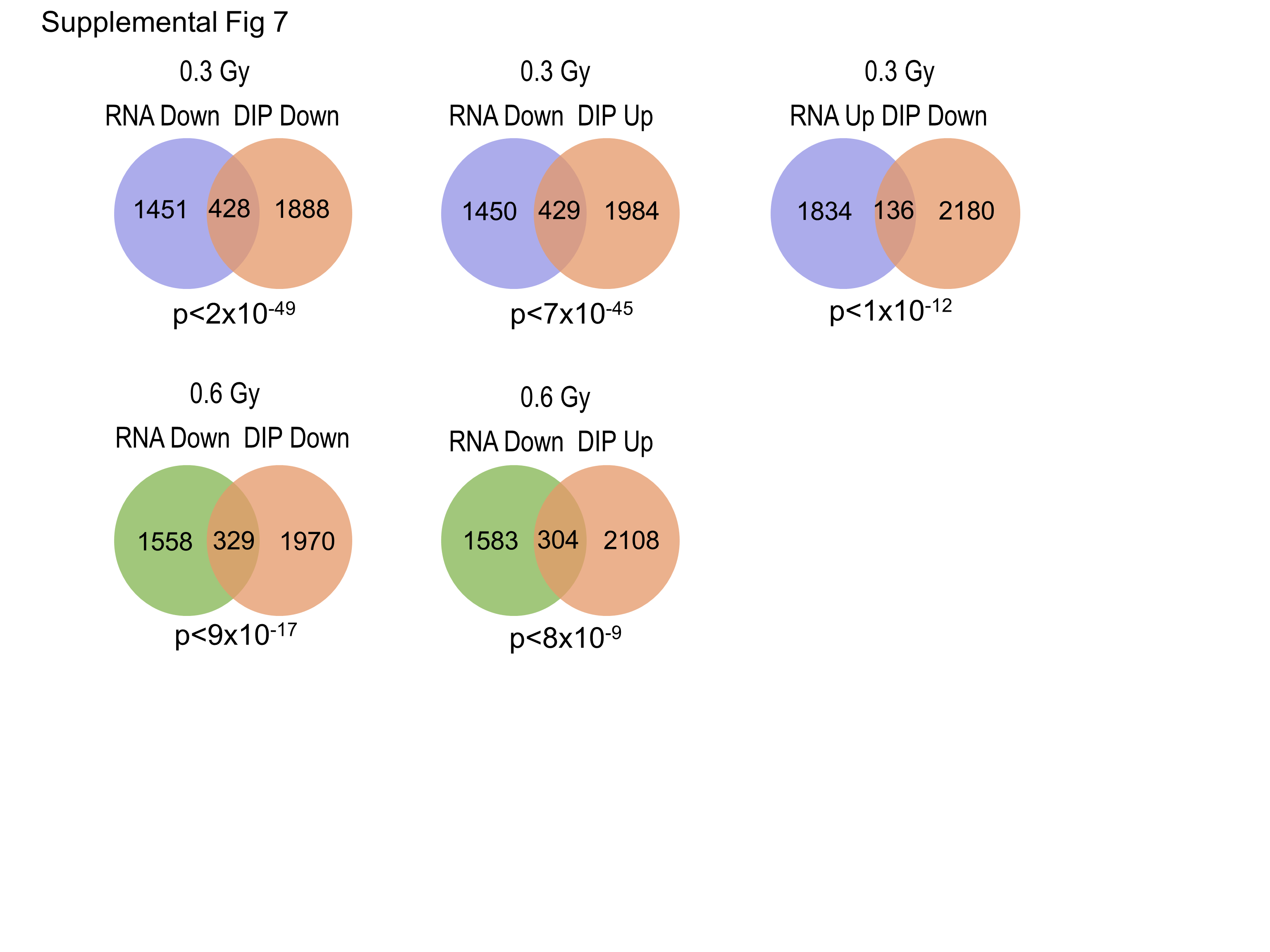

Supplement: Supplementary file 9 [file Image7.TIFF]
